# Heterologous Expression of the *Pyrenophora tritici-repentis* Effector Proteins ToxA and ToxB, and the Prevalence of Effector Sensitivity in Australian Cereal Crops

**DOI:** 10.3389/fmicb.2019.00182

**Published:** 2019-02-12

**Authors:** Pao Theen See, Elyce M. Iagallo, Richard P. Oliver, Caroline S. Moffat

**Affiliations:** Centre for Crop and Disease Management, School of Molecular and Life Sciences, Curtin University, Bentley, WA, Australia

**Keywords:** protein expression, tan spot, *Pyrenophora tritici-repentis*, necrotrophic effector, ToxA, ToxB, wheat, yellow spot

## Abstract

Here, we evaluate the expression of the proteinaceous effectors ToxA and ToxB, produced by the necrotrophic fungal pathogen *Pyrenophora tritici-repentis*, which confer tan spot disease susceptibility on wheat. These necrotrophic effectors were expressed in two heterologous systems: *Escherichia coli* and *Pichia pastoris*. The *E. coli* SHuﬄe system was demonstrated to be superior to *P. pastoris* in generating high-levels of recombinant proteins that were soluble and stable. In addition, protein extracts from *P. pastoris* induced non-specific chlorosis on wheat, postulated to be caused by co-purified glucanases secreted by the host. Up to 79.6 μg/ml of ToxB was obtained using the SHuﬄe system in the absence of the native signal peptide, whilst the ToxA yield was considerably lower at 3.2 μg/ml. Results indicated that a histidine tag at the ToxA C-terminus interfered with effector functionality. Heterologously expressed ToxA and ToxB were tested on a panel of Australian cereals, including 122 varieties of bread wheat, 16 durum, 20 triticale and 5 barley varieties, as well as common plant model species including tobacco and *Arabidopsis thaliana*. A varying degree of effector sensitivities was observed, with a higher ToxB sensitivity and prevalence in the durum and triticale varieties. ToxB-induced chlorosis was also detected on barley. The heterologous expression of effectors that are easily scalable, will facilitate effector-assisted selection of varieties in wheat breeding programs as well as the investigation of *P. tritici-repentis* effectors in host and non-host interactions.

## Introduction

Plant necrotrophic fungal pathogens are known to secrete a multitude of biomolecules during host colonization. Necrotrophic effectors (NE), previously known as host-selective toxins (HSTs), are one such group of these compounds. NEs interact with the host in an inverse gene-for-gene manner. This interaction is in contrast to the conventional gene-for-gene interaction in which the host recognition of the corresponding NE triggers a susceptible disease interaction (Tan et al., 2010). Host recognition of the corresponding NE (Tan et al., 2010) is a major determinant of disease development in such pathosystems (Friesen et al., 2008; Wang et al., 2014).

The necrotrophic fungus *Pyrenophora tritici-repentis* (abbreviated to Ptr) is the causal agent of tan spot disease of wheat [syn. yellow (leaf) spot], and is closely related to pathogens responsible for barley net blotch (Ellwood et al., 2012). Tan spot is a globally significant disease of wheat, and in Australia causes an estimated $212 million of losses per annum (Murray and Brennan, 2009; Oliver et al., 2016). Ptr produces three known effectors, termed ToxA, ToxB, and ToxC, of which ToxA and ToxB are both proteinaceous in nature, while ToxC remains uncharacterized and is thought to be a metabolite (Effertz et al., 2002). The current classification of Ptr isolates into races is based on the ability of a given isolate to produce a combination of these three effectors (Gamba et al., 1998). However, the race classification has been challenged with the discovery of a number of, as yet unidentified, effectors (Ali and Francl, 2002; Lamari et al., 2003; Moolhuijzen et al., 2018; See et al., 2018).

The interest in effector discovery has gained momentum since the affordability of genome sequencing. With the advent of ‘omic’ technologies, coupled with the development of bioinformatics algorithms for effector candidate gene prediction (Lorrain et al., 2015; Testa et al., 2015; Sonah et al., 2016; Sperschneider et al., 2016; Williams et al., 2016), potential effector candidates can readily be mined from the genome. Multiple expression systems are available to then study protein function, with system preference based on scale-up feasibility, cost, relative expression and purity. The *Escherichia coli* bacterium is most commonly used for heterologous expression, and strains that are routinely used include BL21 and its derivatives under the commercial names Origami and Rosetta (reviewed by Terpe, 2006). However, expression is complicated because many effectors have a high cysteine content which plays an important role in protein conformation and disulphide bond formation. In 2012, an *E. coli* strain was engineered, termed SHuﬄe, capable of handling disulphide bond post-translational modification in the cytoplasm (Lobstein et al., 2012). Recently, Zhang et al. (2017) examined the feasibility of using the SHuﬄe strain to express cysteine-rich effector proteins (containing 6 to 16 cysteine residues) from the biotrophic fungus *Melampsora lini* (AvrP, AvrP123, and AvrP4) and the necrotroph *Parastagonospora nodorum* (SnTox1 and SnTox3). Variability between the levels of total protein expressed in the SHuﬄe strain was recorded, with SnTox1 expressing the highest yields followed by AvrP and AvrP123, and a low yield for SnTox3. By comparing the expression of these effectors in SHuﬄe with BL21, the authors demonstrated that individual effector proteins had preferential oxidizing or reducing environments for optimum expression.

Expression of both Ptr ToxA and ToxB has been demonstrated in either *E. coli* (Ballance et al., 1996; Tuori et al., 2000; Kim and Strelkov, 2007) or *Pichia pastoris* (Martinez et al., 2001; Abeysekara et al., 2010; Virdi et al., 2016). However, SHuﬄe has not been tested for either Ptr effector, and the production of Ptr effector proteins in *E. coli* reported to date requires the laborious refolding of the recombinant protein. Moreover for large scale screening of wheat effector sensitivity, crude (not purified) culture filtrate has only been utilized for ToxB in *P. pastoris* systems. Numerous studies have demonstrated the efficacy of ToxA and ToxB in causing necrosis and chlorosis symptoms, respectively, on sensitive host genotypes (Ciuffetti et al., 1997; Martinez et al., 2001; Figueroa et al., 2015). In our previous study, ToxA sensitivity was generally associated with tan spot disease severity of Australian wheat varieties, and the removal of the sensitivity gene (*Tsn1*) enhanced the host disease resistance, although some exceptions were observed (See et al., 2018). However, relatively few studies have assessed wheat varietal sensitivity to ToxB. A recent study by Tran et al. (2017) showed that around 60% of Canadian wheats were sensitive to ToxA and the spore fluid of a ToxC-producing strain, while a smaller percentage of 24% were found to be sensitive to ToxB. Studies have demonstrated ToxB as a major factor in the development of tan spot disease in hexaploid wheat (Abeysekara et al., 2010; Singh et al., 2010), and ToxB has also been reported to play a prominent role in tan spot disease in durum wheat (*Triticum turgidum*) (Virdi et al., 2016). Understanding the interaction between effectors and the host is important for developing tools to improve varietal resistance.

In view of the interest in effector discovery, it is important to assess the approach of using heterologous expression in evaluating protein function. In this study, we investigated the feasibility of using heterologously expressed ToxA and ToxB proteins in the SHuﬄe expression system, and the eukaryotic expression system *P. pastoris* was included to provide a comparable analysis between the two diverse expression systems. Australian wheats and other crops were then examined for effector sensitivity. The aims of this study were to (1) Assess the viability of using SHuﬄe as a method for Ptr effector expression (2) Investigate the effect of native effector signal peptides in heterologous expression (3) Compare the efficiency of two heterologous systems from different organisms (4) Assess the feasibility of heterologous expression systems for large scale effector protein production and purification, and (5) Use purified effectors to screen wheat germplasm to identify sensitive varieties.

## Materials and Methods

### Construction of a Heterologous ToxA and ToxB Expression Cassette for *E. coli*

The *ToxA* (GenBank accession PTRG_04889) and *ToxB* (GenBank accession PZD27634) genes were cloned into the expression vector pET21a(+) (Novagen). The genes were amplified from *P. tritici-repentis* isolate M4 (Moffat et al., 2014) and DW5 (Friesen et al., 2005), respectively. PtrToxA haplotype H15 (PTRG_04889) is the only PtrToxA haplotype found in Australia (Moolhuijzen et al., 2018), and although it has been shown to induce a weaker necrosis symptom in comparison to SnToxA haplotypes (Tan et al., 2012), PtrToxA:H15 was selected in this study in order to test for tan spot specific reactions. Primers are listed in Supplementary Table S1 for the amplification of both genes with and without the signal peptide sequence. Restriction enzyme sites were engineered to facilitate in-frame cloning. PCR amplification was performed in a 50 μl reaction containing 1 × iProof HF master mix (BioRad), 0.2 μM of each primer and 2 μl of cDNA as template. A touchdown PCR reaction was carried out in an Eppendorf Mastercycler using the following cycles; 98°C for 30 s followed by 7 cycles of 98°C denaturation for 5 s, annealing at 64°C for the first cycle and decreasing by 1°C per cycle thereafter, followed by extension at 72°C for 1 min. The next 40 cycles consisted of 98°C denaturation for 5 s, annealing at 58°C for 30 s and 1 min extension at 72°C followed by a final extension of 1 min at 72°C. The PCR product was gel-purified, digested with *Nde*I and *Xho*I, and ligated into the respective restriction sites of pET21a (+). The resulting construct contained the predicted effector protein fused to a hexahistidine tag at the C-terminus. The ligated product was used to transform *E. coli* SHuﬄe or BL21 (DE3) strains. The sequence of the expression construct was confirmed by Sanger sequencing at the Australian Genome Research Facility (AGRF).

### Construction of a Heterologous ToxA and ToxB Expression Cassette for *P. pastoris*

For the heterologous expression of ToxA and ToxB in *P. pastoris*, both genes were cloned into the expression vectors pGAPZA and pGAPZαA (Invitrogen). PCR fragments containing the coding sequence with and without the signal peptide were obtained as detailed above (Supplementary Table S1). Effector genes with the signal peptide were cloned into the *Sac*II/*Apa*I restriction site of pGAPZA while DNA encoding the mature protein was cloned into the *Eco*RI/*Not*I of pGAPZαA vector, which has an α-factor secretion signal sequence for secretion of the protein. The ligated product was then transformed into Top10 cells and the sequence of the construct was verified by Sanger sequencing. The resulting construct from both vectors contained the predicted effector protein fused to a hexahistidine tag at the C-terminus. The expression cassette plasmid was linearized with *Avr*II or *Bsp*HI restriction enzymes and transformed into *P. pastoris* X33 according to the manufacturer’s instructions. Positive *Pichia* clones were routinely grown on YPD agar with zeocin (100 μg/ml) at 30°C.

### Protein Expression and Purification

Protein expression was performed according to Zhang et al. (2017) with some minor modifications. Briefly, the SHuﬄe strain harboring the expression construct was grown in Terrific Broth medium at 37°C containing 100 μg/ml ampicillin until the cultures reached an OD_600_ of 0.4–0.6. Cultures were induced with β-D-1- thiogalactopyranoside (IPTG) at a final concentration of 100 μM and grown for 18 h at 18°C with agitation at 280 rpm. The cells were harvested by centrifugation at 4°C, 3,214 *g* for 10 min and stored at -80°C. The cell pellet harvested from 100 ml culture was resuspended in 5 ml of his-tag binding buffer (20 mM sodium phosphate buffer, 40 mM Imidazole, 500 mM NaCl, pH 7.4) and the cells were lysed using sonication at 10 s pulses with 10 s intervals for 1 min (Sonopuls HD 3100, Bandelin, Germany). The cell debris was pelleted by centrifugation and the effector protein was purified from the cell extract using HisPur Ni-NTA purification spin columns (Thermo Scientific) by gravity flow according to the manufacturer’s instruction. For the *P. pastoris* system, the X33 strain harboring the expression construct was grown in 100 ml YPD media containing 100 μg/ml of zeocin over 3 days at 28°C with agitation at 250 rpm. Cells were then pelleted by centrifugation and the supernatant was concentrated and buffer exchanged with His-tag binding buffer using centrifugal filter column with 3 kDa molecular weight cut-off (Amicon). Proteins were purified from the supernatant using HisPur Ni-NTA purification spin columns (Thermo Scientific) by gravity flow according to manufacturer’s instruction. Proteins for plant bioassays were dialysed in 20 mM sodium phosphate buffer, pH 7.4 and quantified via the bicinchoninic acid assay (Smith et al., 1985). Purified protein was analyzed using Coomassie blue stained SDS-PAGE and Western blots to confirm presence of expressed protein using a 6x-His Tag Monoclonal Antibody (Invitrogen). Briefly, proteins were loaded with 1X sample buffer containing 15 mM Tris-HCl (pH 6.8), 2% SDS, 10% glycerol and 50 mM DTT, boiled for 10 min at 90°C and resolved on 16% SDS-PAGE tricine gel as described in Tan et al. (2012). For Western blotting, proteins were transferred onto a PVDF immune-blot membrane (Bio-Rad) using Towbin transfer buffer (Towbin et al., 1979) containing 0.1% SDS. Each membrane was blocked with 5% milk in Tris-buffered saline (TBS) buffer (50 mM Tris HCl pH 7.6, 150 mM NaCl) containing 0.1% Tween 20, and washed three times for 5 min each wash followed by incubation with 6x-His Tag monoclonal antibody (Invitrogen). Each membrane was washed three times for 5 min each wash in TBS buffer with Tween 20 and TBS buffer. After washing, membranes were incubated in BCIP/NBT liquid substrate solution (SIGMA) for colorimetric detection. Immunoblot assays were repeated at least two times. Purified proteins were stored in freeze-dried form at -80°C until required for plant infiltrations.

### Effector Bioassays

Bioactivity of the heterologously expressed ToxA and ToxB proteins was evaluated *in planta* using plant bioassays as previously described in Moffat et al. (2014). Testing of protein biological activity was performed with a minimum of three replicates and repeated at least twice. The seeds of the monocot panel were sown in vermiculite while the dicot panel were sown in potting mix (Richgro). All plants were grown in a controlled growth environment (Conviron chamber) under a 12 h photoperiod maintained at 22°C. Details of wheat, durum and triticale varieties selected in this study as shown in Supplementary Table S2. For ToxB concentration assay, protein was diluted in 20 mM sodium phosphate buffer to obtain the desired concentration. A minimum of three leaves from independent seedlings of each variety were infiltrated. For the screening of the wheat commercial varieties, ToxA was infiltrated on first leaves while second leaves were used for ToxB. Since ToxB symptoms take longer to develop, the second leaves were used in the assay to avoid the natural occurrence of senescence on first leaves. ToxA infiltrated plants were scored either as sensitive (necrosis present) or insensitive (no symptoms). ToxB-induced chlorosis symptoms were scored from 0 to 5 scale where 0 = no symptoms, 1 = mild chlorosis, 2 = chlorosis, 3 = very strong chlorosis with or without mild necrosis, 4 = chlorosis with necrosis, 5 = necrosis (Supplementary Figure S1). An increment of 0.5 was used if the intensity of the chlorotic symptom was between two band scores. ToxA and ToxB induced symptoms were recorded at 7 and 10 days post-infiltration, respectively.

## Results

### Heterologous Expression of ToxA and ToxB

Since recombinant protein expression using *E. coli* and *P. pastoris* cells are an option due to relatively low cost and feasibility for upscale, we tested the small-scale expression of ToxA and ToxB. ToxA (PtrToxA haplotype H15) contains 114 amino acids and has a size of 13.2 kDa (Ciuffetti et al., 1997), while ToxB is a smaller protein consisting of 64 amino acids with a predicted size of 6.5 kDa (Martinez et al., 2001; Ciuffetti et al., 2010). In order to test the efficacy of these expression systems to process secretion signal peptides, the ToxA and ToxB genes were cloned with and without their respective signal peptides (SP) in both systems.

#### Analysis of Recombinant ToxA and ToxB Proteins in *E. coli* SHuﬄe

To facilitate purification using standard metal ion affinity chromatography (IMAC), proteins were expressed as hexahistidine-tagged. Recombinant proteins of ToxA and ToxB were expressed in SHuﬄe strains under the T7 promoter. Overall, low expression was obtained for constructs containing the SP sequence (Table 1). For ToxA expression, the absence of SP also produced a low protein yield (Table 1). Negligible amount of expressed proteins were detected on the SDS-PAGE, however, Western immunoblotting using a His-tagged monoclonal antibody revealed a single band of the predicted ToxA protein with or without SP of approximately 19.7 and 13.2 kDa (Supplementary Figures S2A,B), respectively. For ToxB containing the SP, recombinant protein appeared to migrate as a double band very close to the predicted size of 8.9 kDa (Supplementary Figure S2C). In an attempt to separate these two bands, fast liquid chromatography (FPLC) was performed. However, due to the relatively low protein yield and the close band proximity, the two bands could not be separated, and therefore were not protein sequenced.

**Table 1:** Protein yield of heterologously expressed ToxA and ToxB in *E. coli* SHuﬄe and *P. pastoris* system in the presence and absence of the native signal peptide sequence.

Heterologous expression	Protein yield (μg) per
system	ml volume of culture
**ToxA**	
*E. coli*	
+ signal peptide (pET21)	3.3
- signal peptide (pET21)	3.2
*P. pastoris*	
+ signal peptide (pGAPZ)	–
- signal peptide (pGAPZα)	1.2
**ToxB**	
*E. coli*	
+ signal peptide (pET21)	6.9
- signal peptide (pET21)	79.6
*P. pastoris*	
+ signal peptide (pGAPZ)	13.1
- signal peptide (pGAPZα)	69.8

In the absence of the SP sequence, ToxB was expressed at approximately the predicted size of 6.5 kDa (Supplementary Figure S2D). The yield obtained from this construct was 79.6 μg per ml culture, which is 10-fold higher than the construct containing the native SP when inducted under similar conditions (Table 1). The higher expression level can be clearly seen on the SDS-PAGE gel with a distinct protein band detected on the induced crude sample. When the gel was immunoblotted with the His-tag antibody, a larger band with weaker intensity at around 14 kDa was also detected along with the predicted ToxB band (Supplementary Figure S2D). Both visible SDS-PAGE bands were excised for protein identification (Supplementary Figure S2E), and mass spectrometry identified both bands as ToxB (Supplementary Table S3).

#### Analysis of Recombinant ToxA and ToxB Proteins in *P. pastoris*

Two different *Pichia* expression vectors (pGAPZA and pGAPZαA) were employed to express ToxA and ToxB in the X33 *P. pastoris* strain under constitutive expression of the glyceraldehyde-3-phosphate (GAP) promoter. Both effectors were cloned with their predicted SP into pGAPZA, and into pGAPZαA in the absence of the signal peptide which contains the α-factor secretion signal. Expression of ToxA in both vectors produced a low yield in comparison to SHuﬄe, with an undetectable amount of protein for the pGAPZA-ToxA construct and 1.2 μg of protein per ml culture for pGAPZαA-ToxA (Table 1). Immunoblots showed that both constructs expressed proteins of the correct predicted molecular weight, however, additional bands were also observed (Supplementary Figures S3A,B). Mass spectrometry protein identification was not performed for *Pichia* ToxA expression due to the low protein yield.

For the *Pichia* expression of ToxB, no protein was detected for the pGAPZA-ToxB construct via SDS-PAGE after IMAC purification, while the immunoblot detected multiple bands with a stronger intensity around 14 kDa (Supplementary Figure S3C). The expressed ToxB protein with the alpha factor secretion signal migrated at the molecular size of approximately 12 kDa (Supplementary Figure S3E), which is larger than the predicted size. The band was observed in both the crude *Pichia* supernatant and purified protein samples. The alpha version also produced a higher protein yield in comparison to the pGAPZA-ToxB construct (Table 1), however, this was lower than the SHuﬄe expression system. Due to the multiple bands observed on the immunoblots for both ToxB constructs, concentrated purified proteins SDS-PAGE bands were excised and the proteins were sequenced. The 14 kDa pGAPZA-ToxB protein band (Supplementary Figure S3D) and four protein bands at approximately 20, 14, 13, and 12 kDa for the alpha vector construct (Supplementary Figure S3F) were identified by protein mass spectrometry. The band from pGAPZA-ToxB was confirmed as ToxB, meanwhile the ToxB protein along with cell wall protein glucanases were detected for the four protein bands from the alpha construct (Supplementary Table S4).

### Bioactivity of Recombinant *P. tritici-repentis* Effectors Protein

To assess the biological activity of the expressed recombinant protein, the expressed ToxA and ToxB proteins from both heterologous systems were affinity His-tagged purified and infiltrated into the leaves of wheat differential lines with known effector sensitivities.

#### Purified His-Tagged ToxA Was Inactive in SHuﬄe and *P. pastoris*

Purified ToxA from both SHuﬄe clones (with the presence or absence of the SP) did not induce any symptoms on the ToxA sensitive line Glenlea when infiltrated even at a high concentration of 200 ng/μl (data not shown), which indicated that the ToxA protein was not active. Similar to the SHuﬄe expression system, ToxA expressed as a fusion protein with a His-tag in *Pichia* also failed to elicit symptoms on Glenlea (data not shown).

#### Removal of His-Tag Restored ToxA Bioactivity

**FIGURE 1:**
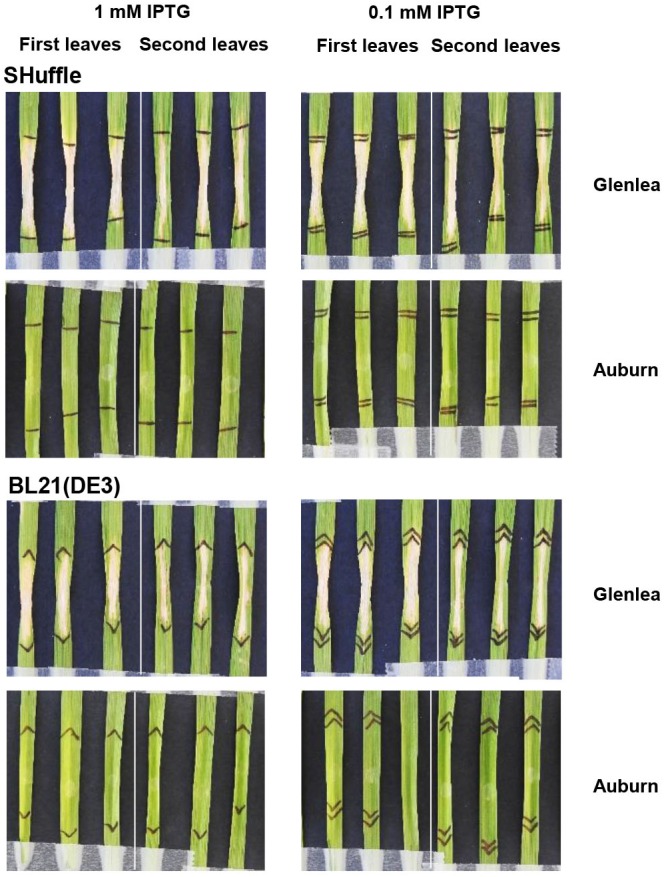
Necrotic symptoms on ToxA differential wheat line Glenlea induced by ToxA present in the *E. coli* crude cell extract. Expression of ToxA was induced with 0.1 and 1 mM IPTG as described in experimental procedures. The ToxA insensitive wheat line Auburn was infiltrated to test for specificity. Representative first and second leaves are shown as indicated for each panel.

To investigate this further, the *ToxA* gene was expressed in another *E. coli* strain BL21 (DE3), a widely used bacterial strain for recombinant protein expression that was previously used for an isoform study of Ptr and *Pa. nodorum* ToxA (Tan et al., 2012). The expression cassette described in Tan et al. (2012) contained the coding region of ToxA and a small portion of the N-terminus of the signal peptide with the predicted MW of 19.25 kDa. In this previous study, biologically active ToxA protein was obtained through an additional refolding step of the inclusion fraction (Tan et al., 2012); however, in order to evaluate the post-translational efficiency between the different expression systems, the soluble fraction of this clone was used to purify the ToxA protein herein. Protein gel analysis of the Tan et al. (2012) His-purified ToxA, when expressed in BL21, showed multiple bands with a distinct pattern of very closely migrated double bands at the estimated size of <15 kDa and approximately 20 kDa (Supplementary Figure S4). Intriguingly, no symptoms were observed when the purified protein was infiltrated on Glenlea at the concentration of 200 ng/μl (data not shown). To rule out any interference that may be caused by the His-terminal tag, a stop codon was inserted in the construct to disrupt the translation of the C-terminal histidine tag. The crude cell extract was then used for the bioassay, since the protein could not be purified using IMAC. The ToxA protein expressed in the absence of the His-tag induced the necrosis on Glenlea (Figure 1). No differences in ToxA activity was observed between the different concentrations of IPTG induced crude cell extract of *E. coli* host BL21. The Tan et al. (2012) construct (with a stop codon) was also transformed into SHuﬄe cells, and similar to BL-21, necrosis was observed following infiltration with crude cell extract (Figure 1). No symptoms were detected on the insensitive wheat line Auburn (Figure 1). These results demonstrate that the histidine tag, when fused to the C-terminus of ToxA protein, interfered with the activity of the recombinant protein, while in the absence of the tag, the ToxA protein was expressed in an active biological state in the soluble fraction.

#### Purified His-Tagged ToxB in SHuﬄe Elicited Genotype-Specific Chlorosis

**FIGURE 2:**
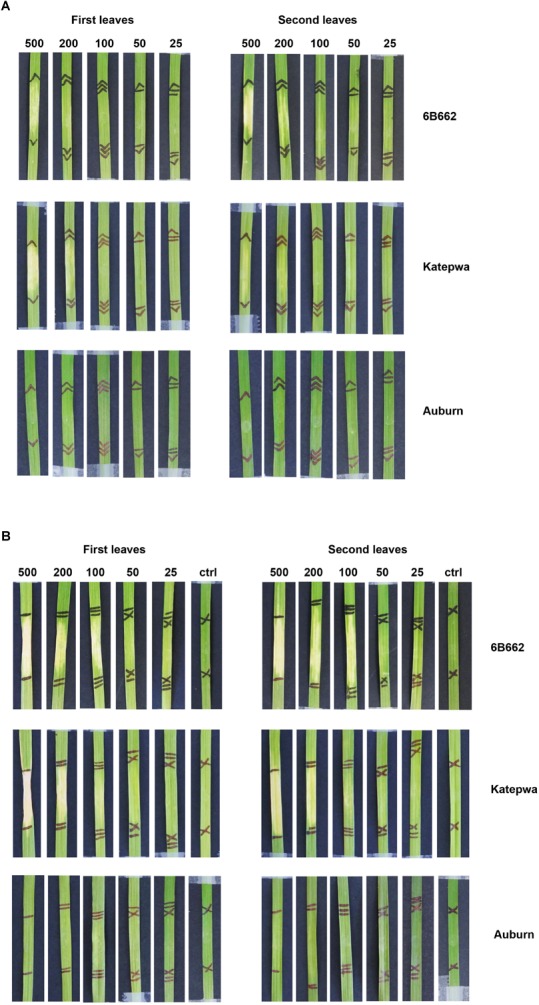
Phenotypes of leaves from the differential wheat lines mediated by the ToxB protein purified from SHuﬄe cells. ToxB expression construct containing the ToxB coding sequence, including the SP **(A)** and the mature protein **(B)**. Bioassay was performed on the first leaves (left panel) and second leaves (right panel). Concentrations of ToxB infiltrated are displayed above the panel. Ctrl, empty vector as control.

For the ToxB activity bioassay, preliminary results showed that individual wheat lines exhibited different sensitivity levels. To evaluate this further, the expressed recombinant ToxB protein was infiltrated at various concentrations ranging from 25 to 500 ng/μl. In the presence of the SP, prominent chlorosis was only observed when infiltrated at 500 ng/μl followed by a milder symptom at the concentration of 200 ng/μl on the ToxB sensitive lines 6B662 and Katepwa (Figure 2A). In contrast, distinct chlorosis on 6B662 was detected following infiltration of the ToxB protein lacking the SP produced in the SHuﬄe strain, at 100, 200, and 500 ng/μl (Figure 2B). Similar concentrations of ToxB (minus SP) also elicited chlorosis on Katepwa with 100 ng/μl of ToxB inducing a less intense chlorosis in comparison to 6B662 (Figure 2B). No chlorosis was observed for the empty vector controls or on the insensitive line Auburn.

Intriguingly, ToxB at the highest concentration also elicited chlorosis on the wheat cultivar 6B365, which is a ToxC differential line (Supplementary Figure S5). However, unlike the distinct chlorotic symptoms observed on the ToxB differential lines, the symptoms on 6B365 were variable and inconsistent between the biological replicates, and occasionally had a streaky necrotic appearances, indicating a non-specific response (Supplementary Figure S5).

#### Purified His-Tagged ToxB in *Pichia* Induced Non-specific Chlorosis

**FIGURE 3:**
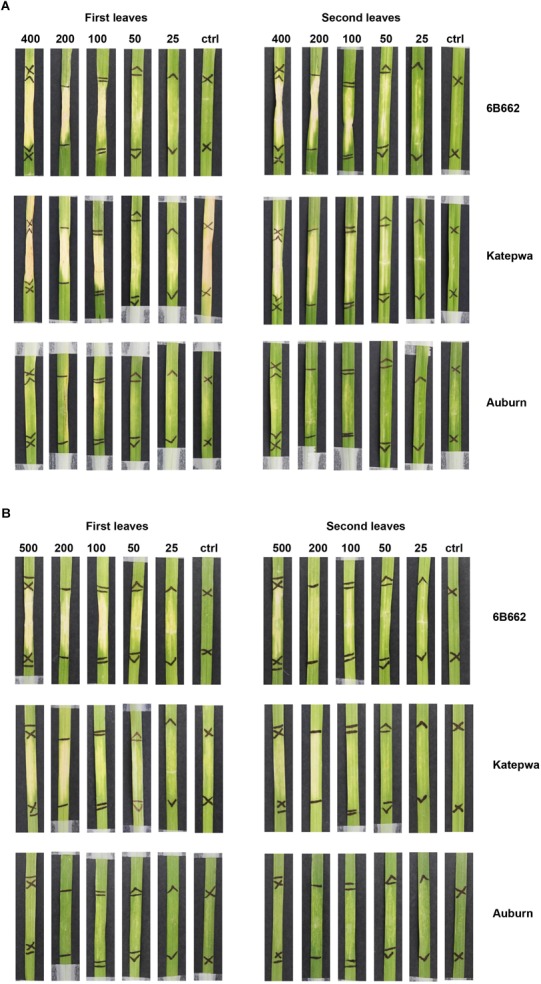
Response of leaves from the differential wheat lines upon infiltration with purified ToxB from X33 *P. pastoris* cultures. ToxB expression construct containing the ToxB coding sequence, including the SP **(A)** and the mature protein **(B)**. Bioassays were performed on the first leaves (left panel) and second leaves (right panel). Concentrations of ToxB infiltrated are displayed above the panel. Ctrl, empty vector as control.

**FIGURE 4:**
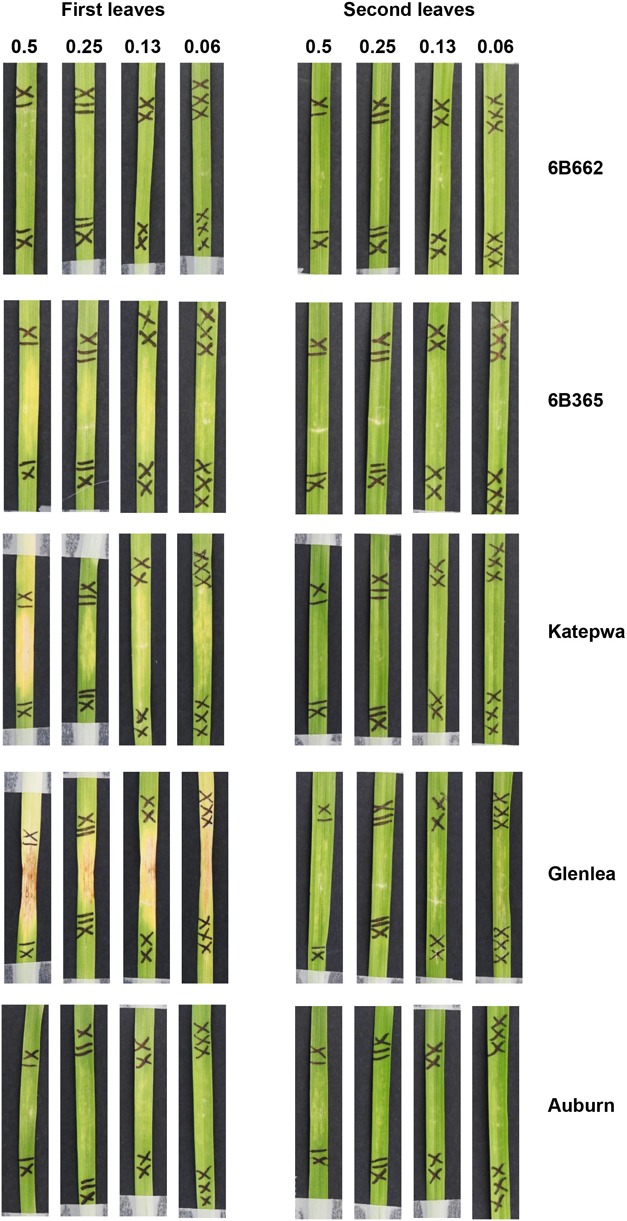
Reactions of leaves of differential wheat lines to the empty vector of the *P. pastoris* expression system. Dilution of the purified empty vector samples is indicated above the panel. Bioassays were performed on the first leaves **(left)** and second leaves **(right)**.

Chlorosis was observed following infiltration of heterologously expressed ToxB purified from the *Pichia* system. Both constructs (pGAPZA-ToxB and pGAPZαA-ToxB) elicited intense chlorotic symptoms, however, these were not restricted to the ToxB sensitive lines (6B662 and Katepwa) (Figure 3A,B). Yellowing was observed on the insensitive line Auburn, most prominently with the native signal peptide present (Figure 3A). Similar symptoms were also observed when the leaves were infiltrated with the empty vector *P. pastoris* control, particularly on the first leaves (Figure 4). Strong symptoms were visible on Glenlea at a dilution of 1/15-fold of the empty vector control, while Katepwa and 6B365 produced mild chlorosis (Figure 4). Altogether these suggest that the intense chlorotic symptoms observed using ToxB protein from the *Pichia* system were attributed to *P. pastoris* background proteins.

### Sensitivity of Commercial Wheat and Non-wheat Cereals to ToxA and ToxB

#### Hexaploid Wheat Varieties

A total of 122 Australian bread wheat varieties were assayed for sensitivity to ToxA and ToxB. ToxA BL-21 cell extract identified 67 varieties (54.9%) as sensitive to ToxA. All varieties rated in the most resistant categories (MRMS and MR) were found to be ToxA insensitive as were most of the intermediate cultivars (Table 2).

**Table 2:** Tan spot disease rating of Australian wheat varieties and their sensitivity to ToxA and ToxB.

	Disease	ToxA	ToxB
Variety	rating^∗^	sensitivity^a^	sensitivity^b^
Corack	MR	-	0.22 ± 0.09
Einstein	MR	-	0.00 ± 0.00
Magenta	MR	-^c^	0.13 ± 0.08
Tennant	MR	-	0.11 ± 0.07
Wyalkatchem	MR	-^c^	0.61 ± 0.11
Adagio	MRMS	-	0.08 ± 0.08
Arrow	MRMS	-	0.17 ± 0.08
Beaufort	MRMS	-	0.11 ± 0.00
Buchanan	MRMS	-	0.00 ± 0.00
Cobra	MRMS	-^c^	0.38 ± 0.10
Cosmick	MRMS	-^c^	0.22 ± 0.09
DS Pascal	MRMS	-	0.17 ± 0.17
EGA Bonnie Rock	MRMS	-^c^	0.21 ± 0.17
Emu Rock	MRMS	-	0.17 ± 0.11
Forrest	MRMS	-	0.00 ± 0.00
GBA Hunter	MRMS	-	0.00 ± 0.00
Hydra	MRMS	-^c^	0.17 ± 0.11
Impress CL Plus	MRMS	-^c^	0.78 ± 0.09
King Rock	MRMS	-^c^	0.00 ± 0.00
Kittyhawk	MRMS	-	0.00 ± 0.00
Mace	MRMS	-^c^	0.50 ± 0.11
Mackellar	MRMS	-	0.00 ± 0.00
Rudd	MRMS	-	0.00 ± 0.00
Scepter	MRMS	-	0.27 ± 0.14
Sunlamb	MRMS	-	0.56 ± 0.10
Sunvex	MRMS	-	0.08 ± 0.08
Tenfour	MRMS	-	0.00 ± 0.00
Yenda	MRMS	-	0.00 ± 0.00
Zen	MRMS	-^c^	0.72 ± 0.12
B53	MS	+	0.08 ± 0.08
Crusader	MS	-	0.00 ± 0.00
Dart	MS	-^c^	0.00 ± 0.00
Gauntlet	MS	-^c^	0.27 ± 0.08
Hartog	MS	+	0.00 ± 0.00
Lancer	MS	+^c^	0.00 ± 0.00
Mansfield	MS	+	1.75 ± 0.17
Mitch	MS	-	0.00 ± 0.00
Naparoo	MS	+	0.00 ± 0.00
Perenjori	MS	-	1.08 ± 0.15
Petrel	MS	+	0.17 ± 0.08
Scenario	MS	-	1.00 ± 0.41
Sentinel	MS	+	0.36 ± 0.10
SQP Revenue	MS	+	0.67 ± 0.08
Strzelecki	MS	-	0.94 ± 0.15
Sunbri	MS	+	0.17 ± 0.17
Sunmax	MS	+	0.00 ± 0.00
Supreme	MS	-^c^	0.00 ± 0.00
Wylah	MS	+	0.08 ± 0.08
Annuello	MSS	+^c^	0.00 ± 0.00
Arrino	MSS	-	0.33 ± 0.17
Barham	MSS	+	0.00 ± 0.00
Beckom	MSS	+	0.00 ± 0.00
Calingiri	MSS	-^c^	0.57 ± 0.12
Carnamah	MSS	-^c^	0.27 ± 0.08
Catalina	MSS	+	0.00 ± 0.00
Clearfield Wht Stl	MSS	+	0.00 ± 0.00
Cobalt	MSS	-	0.00 ± 0.00
Coolah	MSS	+	0.00 ± 0.00
Cutlass	MSS	+	0.00 ± 0.00
Diamondbird	MSS	+	0.00 ± 0.00
EGA Burke	MSS	+	0.00 ± 0.00
EGA Kidman	MSS	+	1.68 ± 0.44
EGA Wedgetail	MSS	+	0.00 ± 0.00
Estoc	MSS	+^c^	0.20 ± 0.10
Flanker	MSS	+	0.00 ± 0.00
Gazelle	MSS	+^c^	0.00 ± 0.00
Giles	MSS	+	0.00 ± 0.00
Impala	MSS	-^c^	0.00 ± 0.00
Kennedy	MSS	-^c^	0.00 ± 0.00
Kord CL Plus	MSS	+	0.08 ± 0.07
Lang	MSS	+	0.00 ± 0.00
Livingston	MSS	-	0.11 ± 0.11
Merinda	MSS	-	0.00 ± 0.00
Longreach Orion	MSS	+	0.00 ± 0.00
QAL 2000	MSS	+	0.00 ± 0.00
QALBIS	MSS	+	0.00 ± 0.00
Sapphire	MSS	+	0.17 ± 0.08
Shield	MSS	+^c^	0.83 ± 0.22
Spitfire	MSS	+^c^	0.00 ± 0.00
Sunguard	MSS	+^c^	0.00 ± 0.00
Sunstate	MSS	+	0.08 ± 0.08
Suntop	MSS	-^c^	0.39 ± 0.07
Sunvale	MSS	+	0.00 ± 0.00
Trojan	MSS	+	1.21 ± 0.32
Viking	MSS	+	0.00 ± 0.00
WAAGAN	MSS	-	0.00 ± 0.00
Wallup	MSS	-^c^	0.36 ± 0.07
Wedin	MSS	+	0.38 ± 0.11
Westonia	MSS	-^c^	0.17 ± 0.11
Sunzell	MSS	+	0.00 ± 0.00
Axe	S	+^c^	0.00 ± 0.00
Bowie	S	+	0.00 ± 0.00
DS Darwin	S	-	0.00 ± 0.00
EGA Eagle Rock	S	-^c^	0.21 ± 0.15
EGA Gregory	S	+^c^	0.00 ± 0.00
EGA Wylie	S	+^c^	0.00 ± 0.00
Elmore CL Plus	S	+	0.08 ± 0.08
Endure	S	+	0.00 ± 0.00
Envoy	S	+	0.00 ± 0.00
Eradu	S	+	0.00 ± 0.00
Grenade CL plus	S	+^c^	0.00 ± 0.00
Halberd	S	+	0.06 ± 0.06
Harper	S	+^c^	0.00 ± 0.00
Hatchet CL plus	S	+	0.00 ± 0.00
Jade	S	-	0.44 ± 0.13
Janz	S	+	0.00 ± 0.00
Justica CL Plus	S	+	0.39 ± 0.14
Kellalac	S	-	0.00 ± 0.00
Kunjin	S	+	0.00 ± 0.00
Machete	S	-^c^	0.38 ± 0.11
Merlin	S	+^c^	0.00 ± 0.00
Peake	S	-	0.22 ± 0.09
Preston	S	+	0.17 ± 0.11
Reliant	S	+	0.00 ± 0.00
Rosella	S	+	0.00 ± 0.00
Ventura	S	+	0.08 ± 0.08
EGA Wentworth	SVS	+	0.00 ± 0.00
Frame	SVS	+^c^	0.08 ± 0.08
Phantom	SVS	+^c^	0.50 ± 0.13
Scout	SVS	+^c^	0.00 ± 0.00
Stiletto	SVS	+^c^	0.00 ± 0.00
Yitpi	SVS	+^c^	0.89 ± 0.18

*^∗^Tan spot disease rating were adapted from National Variety Trial (NVT) 2017 data. ^*a*^+, sensitive (necrosis); -, insensitive (absence of necrosis). ^*b*^Mean chlorotic score ± SEM. ^*c*^ToxA sensitivity of wheat cultivars previously reported in See et al. (2018)*.

**FIGURE 5:**
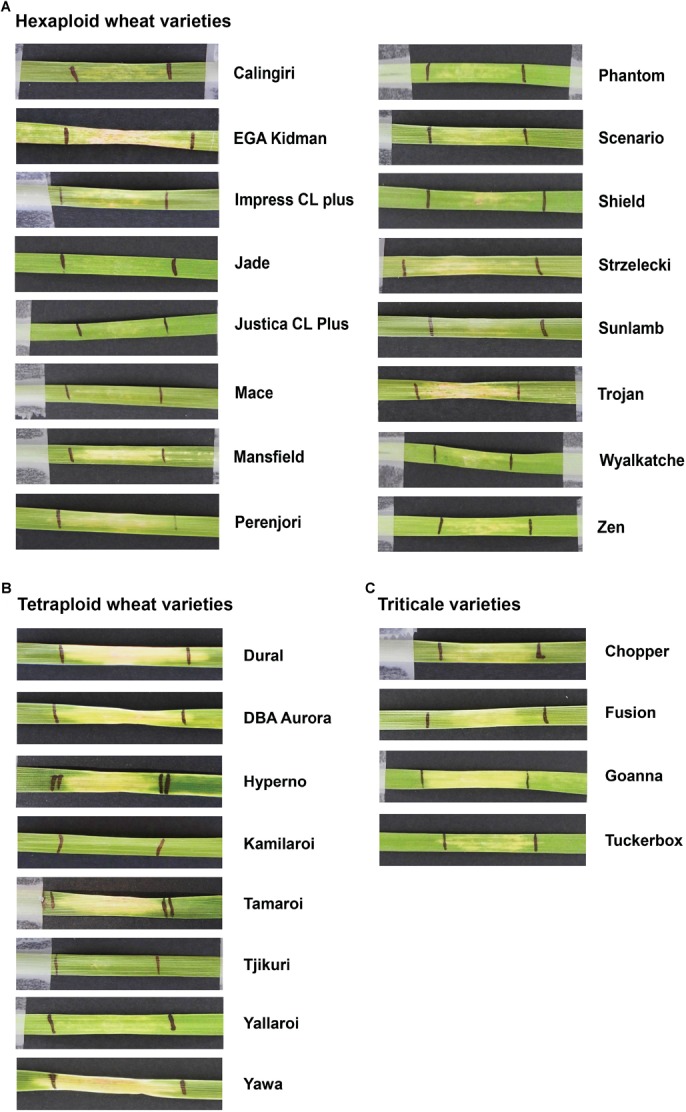
Leaves of Australian commercial cereal varieties exhibiting varying degree of chlorosis upon infiltration with ToxB. Types of cereals: **(A)** hexaploid wheats, **(B)** tetraploid wheats (durum), and **(C)** triticales. Leaves were infiltrated with 200 ng/μl of ToxB protein purified from SHuﬄe cells as described in experimental procedures. Variety names are indicated on the right.

Based on the higher specificity and protein yield exhibited from the SHuﬄe system, ToxB protein expressed without the SP was used to screen the varieties and this was performed on second leaves at 200 ng/μl. Unlike ToxA, which at the concentration used gave a qualitative necrotic or negative reaction, ToxB induced varying degrees of chlorosis depending on the variety tested. Therefore, ToxB symptoms were scored on a scale of 0–5 (Supplementary Figure S1). The majority of the commercial bread wheat varieties assayed were either insensitive to ToxB or produced very mild symptoms (averaging < 1) (Table 2). Only 5 of the 122 varieties produced distinct ToxB chlorosis (mean score ≥ 1.0) (Table 2 and Figure 5A). These were Perenjori, Scenario, Mansfield, EGA Kidman, and Trojan. The latter three were sensitive to ToxA as well.

#### Tetraploid Wheat Varieties

Sixteen durum wheat varieties were also evaluated and 6 (37.5%) were found to be ToxA sensitive and 7 (43.8%) were sensitive to ToxB (Table 3). ToxB-induced chlorosis was notably stronger than that observed in the hexaploid wheats, with six varieties scoring ≥ 2.0 (Table 3 and Figure 5B). These included four varieties that were sensitive to both ToxA and ToxB: Gundaroi, Hyperno, Tjikuri and Yawa. Notably, effector sensitive varieties included those with tan spot disease resistance ratings of MR and MRMS.

**Table 3:** Sensitivity of Australian durum and triticale crops to *P. tritici-repentis* ToxA and ToxB.

Variety	Disease rating^∗^	ToxA sensitivity^a^	ToxB sensitivity^b^
*Durum*			
Arrivato	-	+	0.00 ± 0.00
Caparoi	MR	-	0.00 ± 0.00
DBA Aurora	MR	-	2.56 ± 0.15
Dural	-	-	2.67 ± 0.17
Duramba	-	+	0.00 ± 0.00
EGA Bellaroi	MR	-	0.00 ± 0.44
Gundaroi	-	+	1.08 ± 0.08
Hyperno	MRMS	+	2.00 ± 0.00
Kamilaroi	MRMS	-	0.06 ± 0.06
Saintly	MRMS	-	0.00 ± 0.00
Tamaroi	MRMS	-	2.50 ± 0.26
Tjikuri	MRMS	+	0.17 ± 0.17
Wollaroi	MS	-	0.00 ± 0.00
Yallaroi	MRMS	-	0.50 ± 0.12
Yawa	MR	+	2.50 ± 0.26
Zulu	-	-	2.67 ± 0.17
*Triticale*			
Abacus^∗^PVR^∗^	MR	+	0.00 ± 0.00
Bison	MR	+	0.00 ± 0.00
Chopper	-	-	1.00 ± 0.18
Credit	-	+	1.83 ± 0.17
Eleanor	-	-	0.00 ± 0.00
Everest	MRMS	+	0.00 ± 0.00
Fusion	MR	+	1.20 ± 0.20
Goanna	MR	-	1.25 ± 0.34
Hawkeye	-	-	0.00 ± 0.00
Hillary	-	-	0.00 ± 0.00
Jackie	RMR	-	1.00 ± 0.00
Jaywick	MR	-	0.00 ± 0.00
Kosciusko	-	+	0.00 ± 0.00
Muir	-	-	0.50 ± 0.00
Prime 322	MR	-	1.25 ± 0.11
Rufus	MR	-	1.00 ± 0.00
Tahara	MRMS	-	0.00 ± 0.00
Treat	MR	+	1.17 ± 0.17
Tuckerbox	MR	-	0.75 ± 0.11
Yowie	-	-	0.00 ± 0.00

*^∗^Tan spot disease rating were adapted from National Variety Trial (NVT) 2017 data and Sowing Guide South Australia (2018). ‘-’ Tan spot disease rating was not available. ^*a*^+, sensitive (necrosis); -, insensitive (absence of necrosis). ^*b*^Mean chlorotic score ± SEM*.

#### Triticale Varieties

Twenty varieties of the wheat-rye hybrid triticale were also screened for effector sensitivity. Seven (35.0%) exhibited necrosis following ToxA infiltration, while 8 (40%) scored ≥ 1.0 for ToxB sensitivity (Table 3). Three varieties (Credit, Fusion and Treat) were sensitive to both effectors. Similar to durums, the sensitive triticale varieties rated as MRMS or MR to tan spot (Table 3). The extent of the ToxB-induced chlorosis although higher than the bread wheat, was milder in comparison to the durums (Figure 5C).

#### Sensitivity of Non-wheat Species to ToxA and ToxB

**FIGURE 6:**
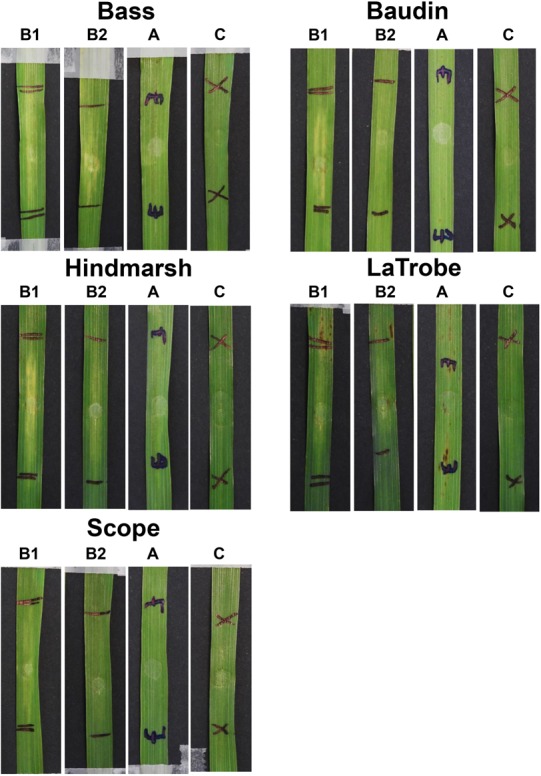
Barley second leaves showing insensitivity to ToxA but displaying varying degree of chlorosis induced by ToxB. Barley leaves were infiltrated with ToxB at the concentration of 500 ng/μl (B1) and 200 ng/μl (B2), and *E. coli* crude cell extract containing ToxA (A). C, empty vector as control.

The sensitivity to ToxA and ToxB was also evaluated on a selection of non-wheat species that included commercial varieties of barley, ryegrass and canola, as well as model dicot and monocot plants such as *Brachypodium distachyon, Arabidopsis thaliana*, and *Nicotiana benthamiana.* Mild chlorosis was observed on barley varieties following ToxB infiltration (Figure 6). At 200 ng/μl, mild chlorosis was observed on Bass, Hindmarsh and LaTrobe while at 500 ng/μl ToxB induced chlorosis on all 5 varieties tested, with Hindmarsh showing the strongest symptoms (Figure 6). No specific symptoms were observed on the other species with either effector (Supplementary Figures S6, S7).

## Discussion

### SHuﬄe a Robust Heterologous Expression System for Ptr ToxB

The utilization of *E. coli* or yeast for heterologous protein production is well documented, but neither Ptr ToxA nor ToxB have been evaluated in these systems. In this study, we compared both expression systems for the two effectors for protein levels, purification ease and bioactivity and demonstrated that the success of obtaining a high-level of biologically active Ptr effector requires a protein-specific approach. One of the disadvantages of using *E. coli* in general for heterologous expression is the inability to carry out post-translational modifications, which is a crucial biological process for protein functionality, including signal peptides. However, the development of the SHuﬄe *E. coli* strain has enabled numerous effector proteins to be expressed successfully (Zhang et al., 2017). Although high cysteine residue counts are usually a concern in effector expression due to the requirement of a reduced environment for disulphide bond formation, the feasibility of expressing effectors does not depend on the numbers of cysteines within the protein molecules as demonstrated in this study. ToxB, which contains four cysteine was amenable in both expression systems tested, with high protein yield obtained via SHuﬄe. In contrast, when a similar expression pipeline was used to express and purify ToxA which has only two cysteines, the protein was found to be inactive. However, the ToxA activity could be restored with the inclusion of a stop codon after the ToxA ORF in the construct, which demonstrated that the C-terminus hexahistidine-tagged fusion interfered with the protein conformation and/or interaction with the host proteins. When Klose et al. (2004) investigated the influence of the position of hexahistidine tagging on the corticotropin-releasing factor (CRF) type 2a mammalian receptor containing 5 cysteines, the *in vitro*-refolded CRF protein expressed in BL21(DE3) displayed different disulphide patterns depending on the position of the His-tag (N or C-terminus). The observed conformational changes, however, had no effect on the expression level and binding affinity. Conversely, in another expression study, functionality of alkaloid biosynthesis enzyme tropinone reductase has been reported to be impaired when a histidine tag was fused to the C-terminus but not at the N-terminus (Freydank et al., 2008). Using molecular modeling approach, they reported a subtle interference of the C-terminal affinity tag at the active site caused by steric or electrostatic interactions. The disruption of the ToxA activity may also suggest that the ToxA C-terminal domain is essential for protein activity, which has not been previously reported while the N-domain has been shown to be required for full ToxA activity (Tuori et al., 2000). Incorporating a His-tag is also known to cause occasional problems such as inappropriate dimer formation (Wu and Filutowicz, 1999). This may explain the occasional doublet or multiple bands that were observed in the expressed proteins.

### Ptr Effectors Are Expressed as Soluble Proteins in the Cytoplasm of SHuﬄe

Previous studies have described heterologous expression of ToxA as both a soluble (Ballance et al., 1996) and insoluble protein in *E. coli* (Tuori et al., 2000; Tan et al., 2012). In this study, the active ToxA expressed without the His-tag was present in the soluble fraction of the *E. coli* cell extract. In both the SHuﬄe and BL21 expression systems, the crude cell extract used in the bioassay was able to induce necrosis on the ToxA sensitive line, indicating that the ToxA protein was expressed in its correct conformation state. In the case of ToxA, it is unknown whether the refolding of the ToxA is a prerequisite for the histidine-tagged fusion protein to obtain bioactivity. Functional ToxA proteins reported to date involve the refolding of the protein after purification. The interference of the hexahistidine tag in ToxA heterologous expression will be useful to investigate further using different expression constructs, such as N-terminal His-tag and on-column cleavage of His-tagged fusion proteins.

In a parallel experiment, active and abundant ToxB protein with the correct predicted size was detected in the soluble fraction of the SHuﬄe expression system. The small trace of the ToxB protein that migrated slower on the reducing gel may be the result of a different conformation, possibly due to intermediate protein assembly or post-translational modification that leads to changes to the electrophoretic mobility. Interestingly, a comparative study on ToxB and the inactive toxb form using NMR demonstrated that conformation changes in the vicinity of one of the cysteine residues allows accessibility to binding partners and increases flexibility of the structure (Nyarko et al., 2014). These heighten the propensity of the protein to be reduced and in the case of toxb, resulted in two protein bands, which were similar to the molecular size recorded in this study estimated from reducing SDS PAGE (Supplementary Figure S2E). Similar to ToxA, overexpression of ToxB in *E. coli* has also been previously reported to give product in the insoluble fraction (Kim and Strelkov, 2007). *In vitro* refolding was necessary to produce the active protein when ToxB was expressed in BL-21 AI^TM^ *E. coli* under the araBAD promoter. In this study, ToxB expressed in SHuﬄe was easily purified using IMAC affinity purification and yield obtained was approximately 80 mg/L of culture, which is 16 times the yield (5 mg/L) reported in the BL-21 strain.

### The Native Signal Peptide Is Detrimental on Protein Yield

In most cases, predicted effectors contain signal sequences at the N-terminus which may complicate the recombinant protein expression. Although the secretion capacity of a heterologous expression system with the native eukaryote signal peptide cannot be inferred in the SHuﬄe system, the signal peptide sequence of Ptr effectors was included in the constructs to allow direct comparison from a methodological perspective. In this study, it was evident that the fusion of the naturally occurring signal peptide had a deleterious effect on the yield of recombinant protein in SHuﬄe cells. It has been reported that eukaryotic signal peptides can effectively replace native signal peptides of *E. coli* for efficient protein expression in a heterologous system. However, optimization of codon usage is required for proper translation (Humphreys et al., 2000). In contrast, a signal peptide that is not compatible can lead to a less stable protein that may also have a higher propensity to aggregate in solution (Singh et al., 2013). This could perhaps explain the observed lower yield and double bands of the expressed ToxB protein containing the signal sequences. It will be worth investigating the feasibility of developing higher eukaryotic signal peptides to function efficiently in *E. coli* secretion machinery for the expression of fungal effectors.

### Host Response to ToxB Is Dose and Genotype Dependent

When the SHuﬄe ToxB protein was assayed for activity on a set of wheat differential lines, the degree of chlorosis was found to be dose dependent and vary with the wheat genotype. For example, the ToxB sensitive lines 6B662 and Katepwa produced different levels of chlorosis when infiltrated with the same concentration of ToxB.

A study by Amaike et al. (2008) showed that the expression level of the *ToxB* gene was linked to the virulence of race 5 Ptr isolates, with higher degrees of *ToxB* expression detected in the more virulent isolates (Amaike et al., 2008). Although the underlying mechanism is not well understood, the regulation of *ToxB* gene and copy number have been suggested to play a role. Such an observation is in line with our bioassay results that showed ToxB sensitivity is regulated in a dose-dependent manner, with higher levels of ToxB eliciting more severe symptom, and this interaction appears to be genotype specific.

Intriguingly, very high ToxB concentrations of 500 ng/μl were able to elicit symptoms on the ToxC differential line 6B365. In contrast, ToxB differential line 6B662 gave clear symptoms with 100 ng/μl. Furthermore, the symptoms on 6B365 were inconsistent with patchy chlorotic or necrotic appearances in contrast to the solid chlorotic symptoms induced on the ToxB differential line, which may suggest a different interaction is at play (Supplementary Figure S5).

### Secreted Endogenous *Pichia* Proteins Induced Non-specific Chlorosis on Wheat

ToxB purified from *Pichia* displayed a lack of cultivar specificity with symptoms observed on the ToxB insensitive variety Auburn. Distinct strong chlorosis was also observed following infiltration of the empty vector control X33 strain on Glenlea, likely due to the secreted endogenous proteins that were co-purified along with ToxB. One of the claimed advantages of *P. pastoris* for production of heterologous proteins is the low abundance of naturally secreted proteins. When cultured on glucose, 20 proteins were detected (Mattanovich et al., 2009), and the majority of these were associated with cell-wall degrading enzymes such as glucanases and endochitinase, which was also observed in this study. The presence of such enzymes in *Pichia* culture supernatant perhaps triggered the observed unspecific chlorotic symptoms on the ToxB insensitive lines. Nevertheless, the *Pa. nodorum* effectors SnTox1 and SnTox3, obtained via heterologous expression in *P. pastoris* have been successfully used in the screening for wheat quantitative trait loci (Cockram et al., 2015; Downie et al., 2018). Yet, due to the strong wheat leaf response caused by secreted endogenous yeast proteins, the application of the *P. pastoris* expression system should be limited for use with effectors that elicit strong symptoms under a minimal incubation period.

### Australian Durum Varieties Are Highly Sensitive to ToxB

Sensitivity to ToxB in Australian bread wheats was found to be rare (4%) in contrast to Canada where 24% of their wheat varieties were reported to be sensitive to ToxB (Tran et al., 2017). In North America, ToxB has been shown to play a more important role in tan spot disease of durum wheat (Virdi et al., 2016). In line with this, the strongest and most frequent ToxB-induced chlorosis was observed in Australian durum varieties.

There was no obvious correlation of the Australian durum or triticale tan spot ratings as measured in Australian field trials to ToxB sensitivity. This lack of correlation can be explained by the absence of the *ToxB* gene in the Australian Ptr population (Antoni et al., 2010). Interestingly, ToxA appears to play a less important role in tan spot disease in durum than in bread wheat, as demonstrated by the ToxA sensitivity of resistant varieties. Similarly, tan spot resistant triticale varieties also exhibited ToxA sensitivity, suggestive again of a less important role of ToxA in triticale tan spot disease. This is in contrast to bread wheat, where resistant varieties are mostly insensitive to ToxA. Although ToxA sensitivity was found to be associated with tan spot disease susceptibility in wheat, the role of *ToxA-Tsn1* interaction in tan spot disease development is dependent on the host background (See et al., 2018). While the Australian varieties are evaluated against tan spot pathogen annually, it will be worth screening these commercial varieties against other Ptr races (e.g., ToxB producing strain) to assess the levels of disease resistance on a broader spectrum to tan spot. Identifying broad-spectrum resistance varieties can be employed in breeding programs to reduce the risk of potential yield loss caused by an incursion of other Ptr races and help to elucidate the interaction in the tan spot pathosystem.

### ToxB-Assisted Selection as a Pre-emptive Breeding Strategy

Ptr ToxB producing races (5, 6, 7, and 8) are known to be present in many countries including Canada, United States, Azerbaijan, Algeria, Syria, and Turkey (Lamari et al., 2003; Benslimane et al., 2011; Abdullah et al., 2017; Gamba et al., 2017) and pose a biosecurity risk to Australia. Thus the availability of heterologously expressed ToxB serves as a useful screening tool to quickly identify and remove sensitive wheat genotypes from breeding germplasm. Such an approach was rapidly adopted by the Australian wheat breeding industry for ToxA and this is reflected in a marked reduction in the release of wheat varieties that harbor the *Tsn1* gene since 2009 (Moffat and Santana, 2018; See et al., 2018).

Although ToxB sensitivity is not common in Australian bread wheat, sensitivity was found in a new variety called Trojan that is one of the top yielding wheat varieties in South Australia (Wheat Crop Harvest Report, 2015). The pedigree of Trojan is LPB 00LR000041/Sentinel, and based on our ToxB screening of effector sensitivity, Trojan inherited the ToxB sensitivity from LPB 00LR000041 since Sentinel was shown to be insensitive. Such information is vital for breeding purposes and with the availability of this effector tool, breeders are able to select germplasm that preserves good traits but breed out sensitivity to ToxB.

### ToxB Induces Chlorosis in Australian Barley Varieties

The barley varieties tested in this study all displayed sensitivity to ToxB with chlorosis generally milder than in wheat. This is in contrast to Ali and Francl’s (2001) study in which 12 barley cultivars assayed were insensitive to ToxB. In a more recent study by Aboukhaddour and Strelkov (2016), barley genotypes that produced foliar chlorosis after inoculation with Ptr isolates were shown to be sensitive to ToxB. Closer examination of Ptr infection on barley showed that the fungus was able to penetrate the epidermal cells and proliferate intracellularly on both resistant and susceptible barley genotypes, although no clear distinctions were observed between susceptible and resistant varieties at the mesophyll layer (Aboukhaddour and Strelkov, 2016). The level of ToxB sensitivity, however, was reported to be lower in barley with a minimum concentration of approximately 7 ng/μl required to cause symptoms. In contrast, our study showed that mild chlorosis was only detected when barley leaves were infiltrated at the concentration of 200 ng/μl. It remains to be determined if these Australian barley varieties are susceptible to tan spot disease.

### Insensitivity of Model Plant Species to Ptr Effectors

Recently, functional studies of ‘putative’ effectors have been performed using model plant systems as surrogates to study effector-host interactions. For example, the transient expression of effectors such as Avr2 from the root fungal pathogen *Fusarium oxysporum* f. sp. *lycopersici* (Ma et al., 2012) and candidate effectors from the wheat yellow rust pathogen *Puccinia striiformis* f. sp. *tritici* (Petre et al., 2016) have been introduced into the model plant *Nicotiana benthamiana* as GFP fusion proteins via agroinfiltration. This has proven a valuable tool to study interactions between putative effectors and the host counterparts *in planta*. However, the use of surrogate host model systems may not be universally applicable for all effectors. Besides barley, ToxA and ToxB did not elicit any symptoms on the non-wheat plants tested which included common plant models such as *A. thaliana, B. distachyon* and *N. benthamiana*. The absence of ToxA and ToxB effector sensitivity has also been reported on non-cereal grasses such as wheatgrass, bromegrass and needlegrass (Ali and Langham, 2015). Nevertheless, it is plausible that other Ptr effectors that have yet to be identified may be used in such systems.

## Conclusion

Characterizing new effectors is often challenged by the drawback of expressing recombinant proteins for bioassays, biochemistry and structural biology. In this evaluation of heterologous expression on different host systems, we demonstrated that there is no ‘one fits all’ expression pipeline. Generating active recombinant proteins with optimal yield has to be determined empirically for individual effectors. The production of a high-yield soluble ToxB protein that is easily scalable will be a valuable resource for various applications including screening effector sensitivity in wheat germplasm, studying ToxB-barley interactions and mapping of the wheat ToxB sensitivity *Tsc2* gene.

## Author Contributions

PS wrote the manuscript and performed the plant bioassays. EI constructed the expression cassettes, expressed the proteins, and performed the plant bioassays. RO critically reviewed the manuscript. CM conceptualized and designed the project and reviewed the manuscript. All authors read and approved the final manuscript.

## Conflict of Interest Statement

The authors declare that the research was conducted in the absence of any commercial or financial relationships that could be construed as a potential conflict of interest.
