# Supplementary material for: Heterologous Expression of the Pyrenophora tritici-repentis Effector Proteins ToxA and ToxB, and the Prevalence of Effector Sensitivity in Australian Cereal Crops
Source: Front Microbiol. 2019 Feb 12;10:182. doi: 10.3389/fmicb.2019.00182 (PMC6379657; doi:10.3389/fmicb.2019.00182)

**Table S1.** List of primers used in this study.

| **Primer** | **Sequence (5’ to 3’)** |
| --- | --- |
| ToxA_F*  ToxA-sp_F  ToxA_BL21F*  ToxA_R  ToxB_F*  ToxB-sp_F  ToxB_R  ToxApi_F*  ToxApi_R  ToxApiα_F  ToxApiα_R  ToxBpi_F*  ToxBpi_R  ToxBpiα_F  ToxBpiα_R | GGAATTC**CATATG**TCAATCACAATCAACCC  GGAATTC**CATATG**CGTTCTATCCTCGTACT  AAGGCTC**CATATG**GCCCCAACGCCTGAAGCCG  CCG**CTCGAG**ATTTTCTAGCTGCATTCTCC  GGAATTC**CATATG**GCGCCTATATTCAAGAC  GGAATTC**CATATG**AACTGCGTCGCCAATAT  CCG**CTCGAG**ACAACGTCCTCCACTTTGCA  TCC**CCGCGG**GTCATGCGTTCTATCCTCGTACT  **GGGCCC**ATTTTCTAGCTGCATTCTCC  G**GAATTC**TGCATGTCAATCACAATCAACCC  ATAGTTTA**GCGGCCGC**ATTTTCTAGCTGCATTCTCC  TCC**CCGCGG**AAAATGGCGCCTATATTCAAGAC  **GGGCCC**ACAACGTCCTCCACTTTGCA  G**GAATTC**AACTGCGTCGCCAATATCTT  ATAGTTTA**GCGGCCGC**ACAACGTCCTCCACTTTGCA |

*Forward primer that amplifies signal peptide sequence. Restriction enzyme sites are in bold.

**Table S2**. List of commercial cereal crops used in this study.

| **Wheat (Varieties)** | **Year released** | **Released by / Developed by** |
| --- | --- | --- |
| Adagio SF | 2014 | RAGT seeds |
| Annuello | 2003 | DPI, VIC |
| Arrino | 1997 | DAFWA DPRID |
| Arrow | 2016 | LRPB |
| Axe | 2007 | AGT |
| B53 | 2015 | Elders/Seedmark |
| Barham | 2006 | AGT |
| Beaufort | 2008 | LRPB |
| Beckom | 2015 | AGT |
| Bowie | 1998 | UA |
| Buchanan | 2015 | Austgrains |
| Calingiri | 1997 | DPRID |
| Carnamah | 1996 | DPRID |
| Catalina | 2006 | LRPB |
| Clearfield WHT STL | 2000 | DPRID |
| Cobalt | 2015 | Seedmark |
| Cobra | 2011 | LRPB |
| Coolah | 2016 | AGT |
| Corack | 2011 | AGT |
| Cosmick | 2014 | InterGrain |
| Crusader | 2007 | LRPB |
| Cutlass | 2015 | AGT |
| Dart | 2012 | LRPB |
| Diamondbird | 1997 | DPI, NSW |
| DS Darwin | 2015 | Advantage wheats Pty Ltd |
| DS Pascal | 2015 | Advantage wheats Pty Ltd |
| EGA Eagle Rock | 2004 | EGA |
| EGA Bonnie Rock | 2002 | EGA |
| EGA Burke | 2006 | EGA |
| EGA Gregory | 2004 | EGA |
| EGA Kidman | 2008 | DAFF QLD |
| EGA Wedgetail | 2002 | EGA |
| EGA Wentworth | 2004 | EGA |
| EGA Wylie | 2004 | EGA |
| Einstein | 2012 | Heritage Seeds |
| Elmore CL Plus | 2011 | AGT |
| Emu Rock | 2011 | InterGrain |
| Endure | 2008 | InterGrain |
| Envoy | 2011 | LRPB |
| Eradu | 1981 | DPRID |
| Estoc | 2010 | AGT |
| Flanker | 2015 | LRPB |
| Forrest | 2011 | Advantage wheats Pty Ltd |
| Frame | 1994 | UA |
| Gauntlet | 2011 | LRPB |
| Gazelle | 2012 | Value Added Wheat CRC / LRPB |
| GBA Hunter | 2003 | Grain Biotech Australia Pty Ltd |
| Giles | 1999 | DAFF QLD |
| Grenade CL plus | 2014 | AGT |
| Halberd | 1969 | UA |
| Harper | 2013 | InterGrain |
| Hartog | 1982 | DAFF QLD |
| Hatchet CL plus | 2015 | AGT |
| Hydra | 2014 | InterGrain |
| Impala | 2011 | Value Added Wheat CRC / LRPB |
| Impress CL Plus | 2015 | InterGrain |
| Jade | 2015 | Elders / Seedmark |
| Janz | 1989 | DAFF QLD |
| Justica CL Plus | 2011 | AGT |
| Kellalac | 1988 | DPI, VIC |
| Kennedy | 1998 | DAF, QLD |
| King Rock | 2009 | InterGrain |
| Kittyhawk | 2017 | LRPB |
| Kord CL Plus | 2011 | AGT |
| Kunjin | 2010 | InterGrain |
| Lancer | 2013 | LRPB |
| Lang | 2000 | DAFF QLD |
| Livingston | 2007 | AGT |
| Mace | 2007 | AGT |
| Machete | 1985 | SARDI |
| Mackellar | 2001 | Advantage wheats Pty Ltd |
| Magenta | 2007 | InterGrain |
| Mansfield | 2010 | Advantage wheats Pty Ltd |
| Merinda | 2007 | AGT |
| Merlin | 2012 | LRPB |
| Mitch | 2014 | AGT |
| Naparoo | 2008 | AGT |
| Orion | 2009 | LRPB |
| Peake | 2007 | Nugrain |
| Perenjori | 1996 | DAFWA |
| Petrel | 1996 | DPI, NSW |
| Phantom | 2012 | LRPB |
| Preston | 2008 | Advantage wheats Pty Ltd |
| QAL 2000 | 2000 | Value added wheat CRC |
| QALBIS | 2002 | Value added wheat CRC |
| Reliant | 2016 | LRPB |
| Rosella | 1985 | DPI, NSW |
| Rudd | 2001 | CSIRO |
| Sapphire | 2003 | GBA |
| Scenario SF | 2014 | RAGT seeds |
| Scepter | 2015 | AGT |
| Scout | 2009 | LRPB |
| Sentinel | 2005 | LRPB |
| Shield | 2012 | AGT |
| Spitfire | 2010 | LRPB |
| SQP Revenue | 2010 | CSIRO |
| Stiletto | 1993 | SARDI |
| Strzelecki | 2000 | DAFF QLD |
| Sunbri | 1990 | University of Sydney |
| Sunguard | 2011 | AGT |
| Sunlamb | 2015 | AGT |
| Sunmax | 2016 | AGT |
| Sunstate | 1993 | University of Sydney |
| Suntop | 2012 | AGT |
| Sunvale | 1993 | University of Sydney |
| Sunvex | 2008 | AGT |
| Sunzell | 2006 | AGT |
| Supreme | 2014 | InterGrain |
| Tenfour | 2015 | Seedmark |
| Tennant | 1998 | CSIRO |
| Trojan | 2013 | LRPB |
| Ventura | 2004 | University of Sydney |
| Viking | 2014 | LRPB |
| WAAGAN | 2008 | EGA |
| Wallup | 2011 | AGT |
| Wedin | 2010 | InterGrain |
| Westonia | 1997 | DAFWA |
| Wyalkatchem | 2001 | InterGrain |
| Wylah | 1999 | DPI NSW |
| Yenda | 2006 | AGT |
| Yitpi | 1999 | SARDI |
| Zen | 2014 | InterGrain |

| **Durum (Varieties)** | **Year released** | **Released by/ Developed by** |
| --- | --- | --- |
| Arrivato | 1999 | Heritage Seeds |
| Caparoi | 2008 | NSW DPI |
| DBA Aurora | 2014 | UA |
| Dural | 1956 | NSW DPI |
| Duramba | 1970 | NSW DPI |
| EGA Bellaroi | 2004 | EGA |
| Gundaroi | 1999 | NSW DPI |
| Hyperno | 2008 | NSW DPI / AGT |
| Kamilaroi | 1983 | DPI NSW |
| Saintly | 2008 | NSW DPI /AGT |
| Tamaroi | 1997 | DPI NSW |
| Tjikuri | 2010 | UA |
| Wollaroi | 1993 | DPI NSW |
| Yallaroi | 1987 | DPI NSW |
| Yawa | 2012 | UA |
| Zulu | 2008 | AGT |

| **Triticale (Variesties)** | **Year released** | **Released by/ Developed by** |
| --- | --- | --- |
| Abacus | 1992 | UA |
| Bison | 2014 | AGT |
| Chopper | 2010 | AGT |
| Credit | 1997 | UA |
| Eleanor | 2001 | University of Sydney University |
| Everest | 1999 | University of New England |
| Fusion | 2012 | AGT |
| Goanna | 2011 | Cooper & Elleway |
| Hawkeye | 2007 | AGT |
| Hillary | 2000 | University of Sydney University |
| Jackie | 1999 | University of Sydney |
| Jaywick | 2007 | AGT |
| Kosciusko | 2002 | University of New England |
| Muir | 1988 | DPRID |
| Prime 322 | 2001 | University of Sydney |
| Rufus | 2005 | University of New England |
| Speedee | 2002 | UA |
| Tahara | 1987 | DPI, VIC |
| Treat | 1998 | UA |
| Tuckerbox | 2009 | Seed Distributors |
| Yowie | 2010 | Cooper & Elleway |

DPI VIC = Department of Primary Industries Victoria, AGT = Australian Grain Technologies, DPRID = Department of Primary Industries and Regional Development (formerly known as DAFWA Agriculture and Food, Western Australia), GBA = Grain Biotech Australia, EGA = Enterprise Grains Australia, UA= University of Adelaide, DAF QLD = Department of Agriculture and Fisheries, Queensland, SARDI = South Australia Research and Development Institute, LRPB = LongReach Plant Breeders .

**Table S3.** Summary of top protein hits identified from Mascot search of trypsin digested mass spectrometry of SHuffle ToxB protein bands.

| **SDS-PAGE bands** | **Protein hits** | **Accession** | **Score** | **Peptide matches** | **p < 0.05** | **emPAI** |
| --- | --- | --- | --- | --- | --- | --- |
| < 10 kDa | ToxB Host-selective toxin protein | Q9C173 | 920 | 45 | 41 | 1.13 |
|  | 40S ribosomal protein S6 | W3VSW8 | 63 | 1 | 1 | 0.14 |
| ~14 kDa | ToxB Host-selective toxin protein | Q9C173 | 176 | 12 | 7 | 1.13 |
|  | Uncharacterized protein | A0A0F7ZIF8 | 44 | 12 | 1 | 0.06 |

**Table S4.** Summary of top protein hits identified from Mascot search of trypsin digested mass spectrometry of Pichia ToxB protein bands.

| **SDS-PAGE bands** | **Protein hits** | **Accession** | **Score** | **Peptide matches** | **p < 0.05** | **emPAI** |
| --- | --- | --- | --- | --- | --- | --- |
| B1 (~ 14 kDa) | ToxB Host-selective toxin protein | Q9C173 | 59 | 12 | 3 | 0.94 |
| B2 (~ 12 kDa) | Cell wall protein with similarity to glucanases | C4QVL7 | 86 | 4 | 4 | 0.22 |
|  | ToxB Host-selective toxin protein | Q9C173 | 61 | 108 | 13 | 1.13 |
| B3 (~13 kDa) | Cell wall protein with similarity to glucanases | C4QVL7 | 77 | 4 | 4 | 0.19 |
|  | ToxB Host-selective toxin protein | Q9C173 | 60 | 33 | 1 | 0.94 |
|  | Uncharacterized protein | A0A0B4H4F6 | 53 | 5 | 0 | 0 |
| B4 (~ 14 kDa) | Glyceraldehyde-3-phosphate dehydrogenase | A0A1A0HCE0 | 81 | 4 | 2 | 0.19 |
|  | Cell wall protein with similarity to glucanases | C4QVL7 | 73 | 3 | 2 | 0.19 |
|  | ToxB Host-selective toxin protein | Q9C173 | 55 | 30 | 3 | 0.94 |
|  | DNA-binding protein | B7XNL8 | 55 | 1 | 1 | 0.36 |
|  | Uncharacterized protein | A0A0B4H4F6 | 53 | 4 | 0 | 0 |
| B5 (~20 kDa) | Cell wall protein with similarity to glucanases | C4QVL7 | 134 | 10 | 6 | 0.42 |
|  | ToxB Host-selective toxin protein | Q9C173 | 54 | 11 | 1 | 0.94 |
|  | Ferulic acid esterase | SCN69328.1 | 53 | 13 | 1 | 0.17 |

Refer to figure below for the SDS-PAGE protein bands

**Table S4.** Continued


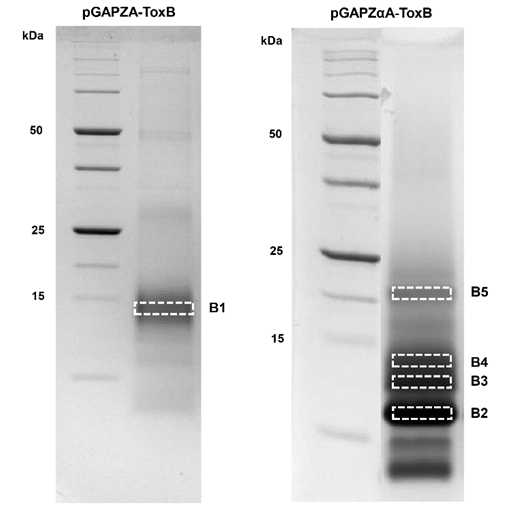

Supplement: Supplementary file 1 [file Table_1.docx]
