# Supplementary material for: Heterologous Expression of the Pyrenophora tritici-repentis Effector Proteins ToxA and ToxB, and the Prevalence of Effector Sensitivity in Australian Cereal Crops
Source: Front Microbiol. 2019 Feb 12;10:182. doi: 10.3389/fmicb.2019.00182 (PMC6379657; doi:10.3389/fmicb.2019.00182)

**Fig. S1.** Representative leaves denoting the scoring scale for ToxB-induced chlorosis. Symptoms were recorded 10 days post-infiltration.


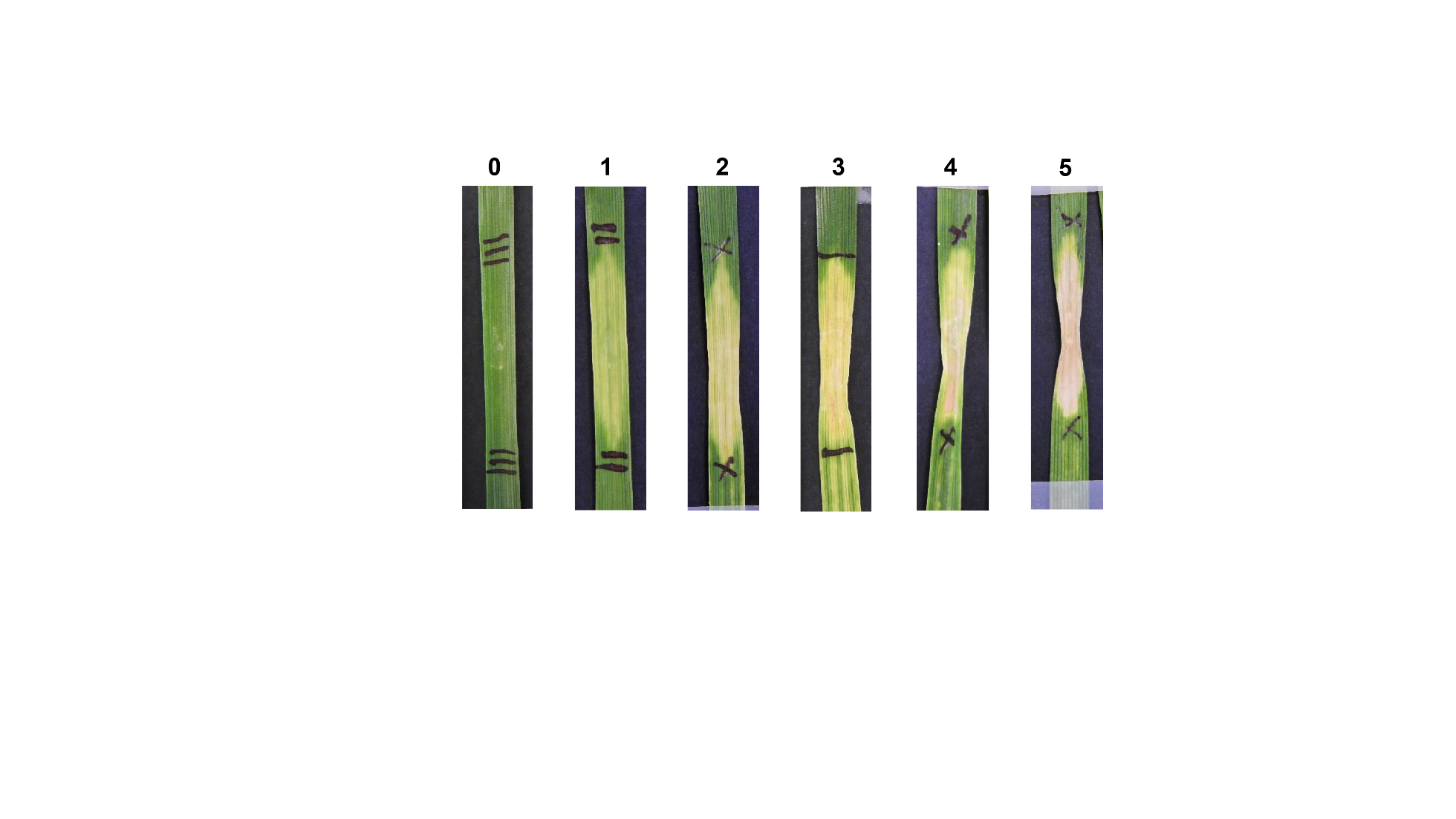


**Fig. S2.** SDS-PAGE gel and Western blot analysis of ToxA and ToxB protein expressions in the SHuffle expression system. Expression construct containing the coding sequence, including the SP (A, C) and the mature protein, without the SP (B, D). Lane 1, *E. coli* cell extract of non-induced sample; Lane 2, induced sample; Lane 3, His-purified protein obtained as described in experimental procedures. E, SDS-PAGE gel of concentrated His-purified ToxB protein (without SP). Arrow denotes protein bands excised for protein mass spectrometry. Molecular weight marker (kDa) is indicated on the left.


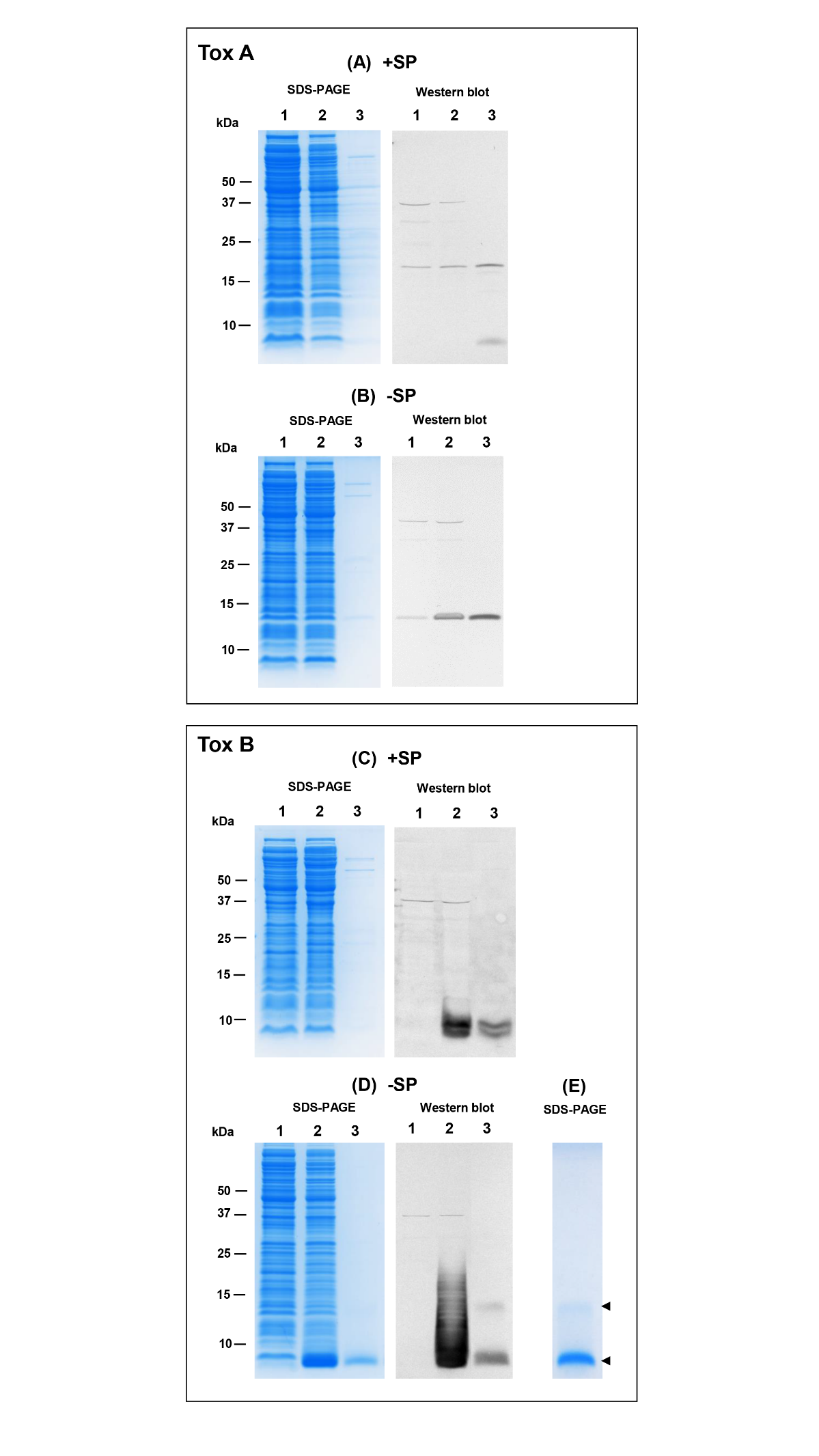


**Fig. S3.** SDS-PAGE gel and Western blot analysis of ToxA and ToxB protein expressions in the *P. pastoris* expression system. Expression construct containing the ToxA coding sequence, including the SP (A) and the mature ToxA protein (B). Left panel, SDS-PAGE gel of His-purified ToxA protein and right panel, Western blot. Expression construct containing the ToxB coding sequence, including the SP (C) and the mature ToxB protein (E). Lane 1, X33 non-purified culture supernatant (100 µg); Lane 2, His-purified protein (10 µg) obtained as described in experimental procedures. SDS-PAGE gel of concentrated His-purified ToxB protein containing SP (D) and without SP (F). Arrow denotes protein bands excised for protein mass spectrometry. Molecular weight marker (kDa) is indicated on the left.


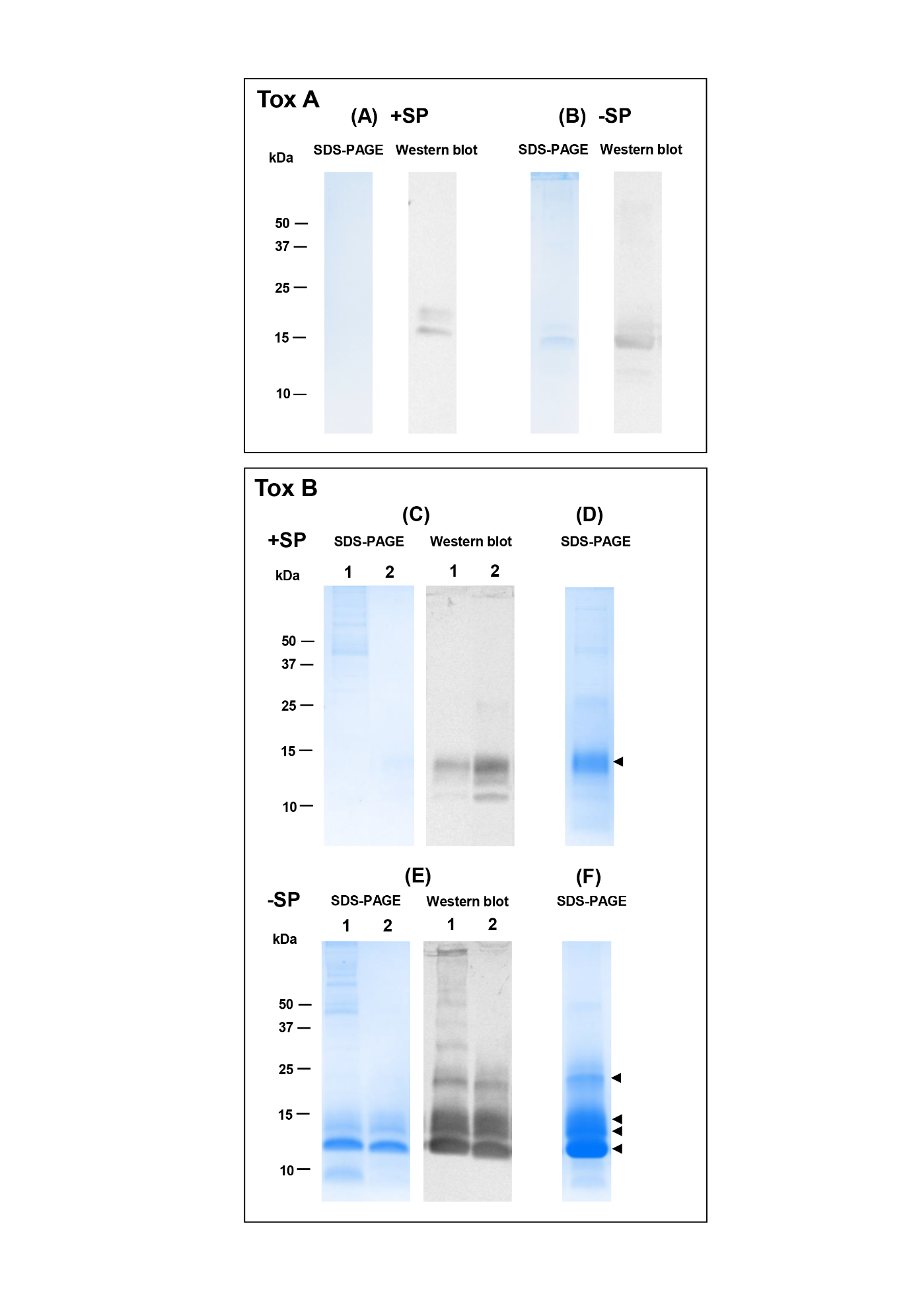


**Fig. S4.** SDS-PAGE and Western blot analysis of ToxA expression containing C-terminal histidine tag in BL21 (DE3). Lane 1, IMAC purified elution of ToxA protein and lane 2, IMAC purified elution of empty vector control. The molecular marker (kDa) is indicated on the left.


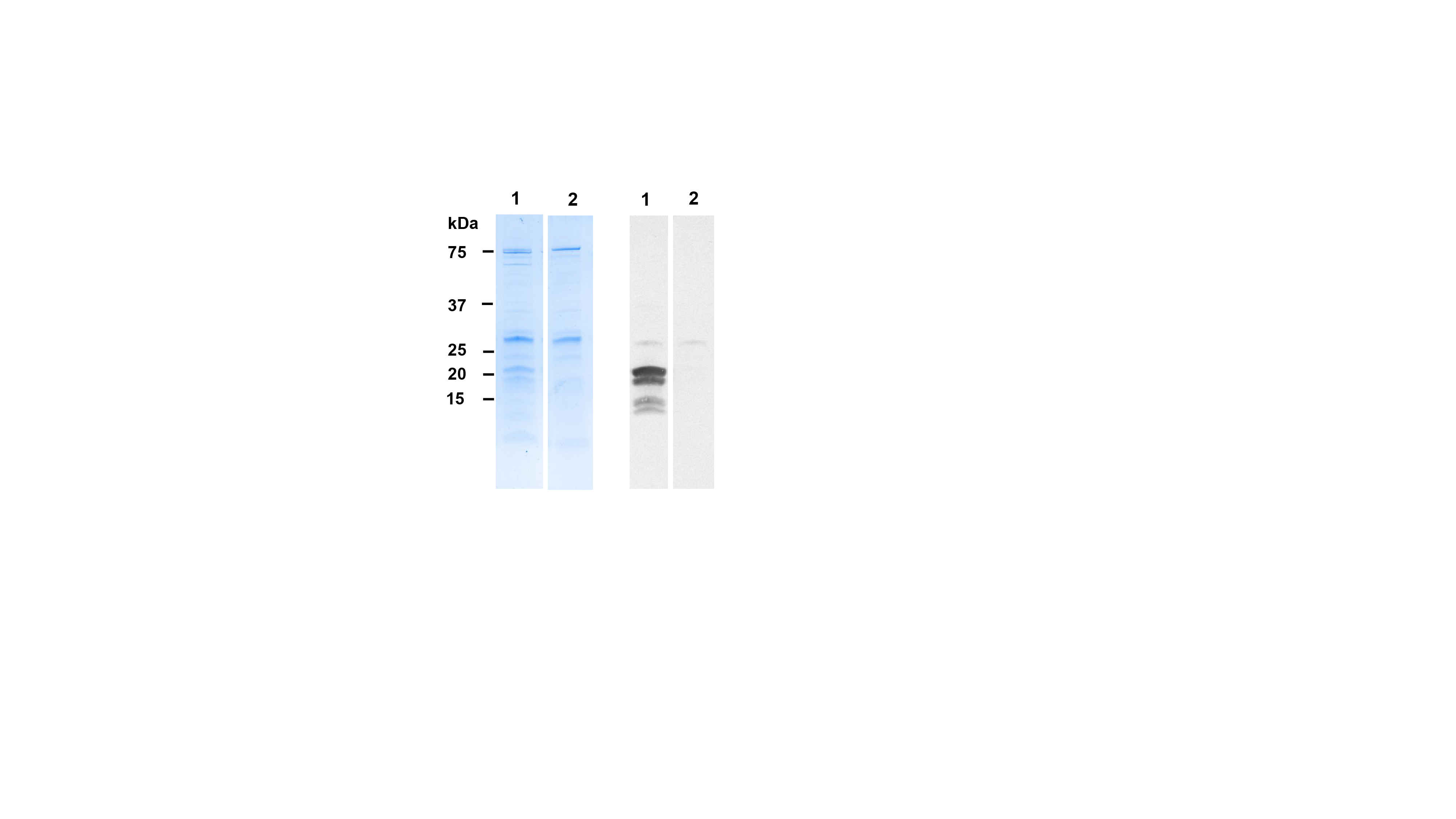


**Fig. S5.** Leaf symptoms displayed by wheat line 6B365 (ToxC differential line) in comparison the distinct chlorotic symptoms induced by ToxB on wheat line 6B662 (ToxB differential line). Leaves were photographed 10 days post-infiltration with 500 ng/µl of ToxB.


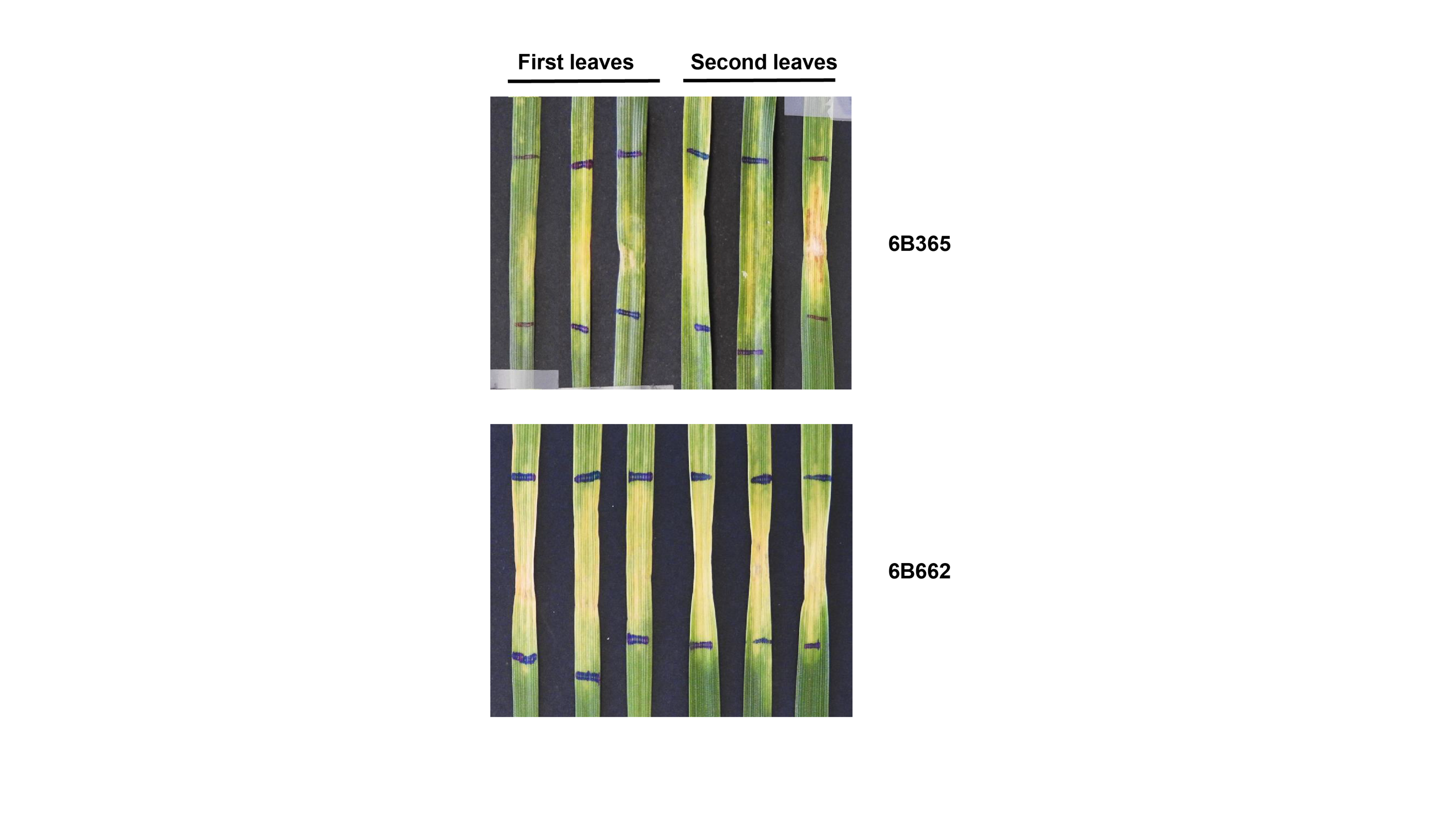


**Fig. S6.** Response of dicot plants to *P. tritici-repentis* effectors. Leaves of (A) *Arabidopsis thaliana*, (B) *Nicotiana benthamiana* (C) and Canola (*Brassica napus L*.) were infiltrated with ToxB at the concentration of 500 ng/µl (B1) and 200 ng/µl (B2), *E. coli* crude cell extract containing ToxA (A) and empty vector as control (C). Representative leaves were photographed 10 days post-infiltration. Scale bar, 10 cm.


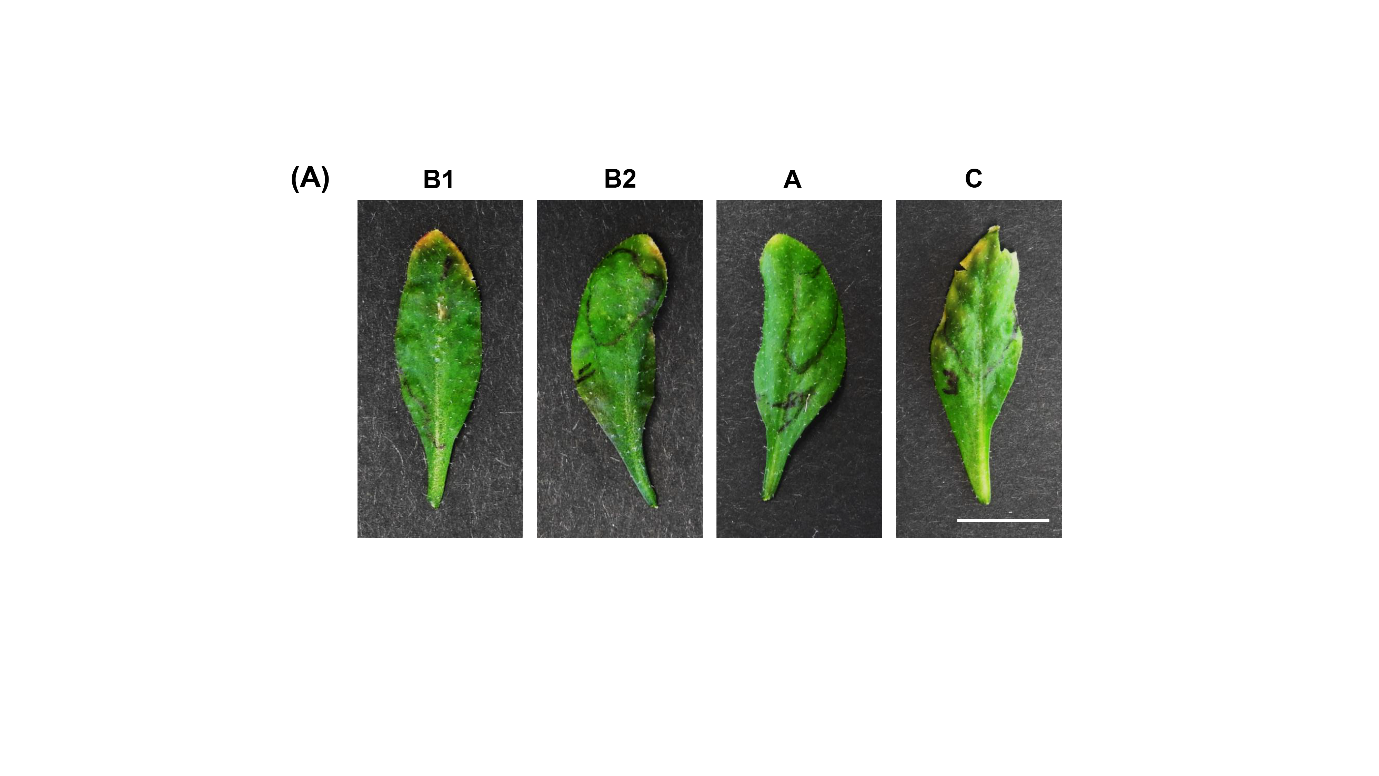


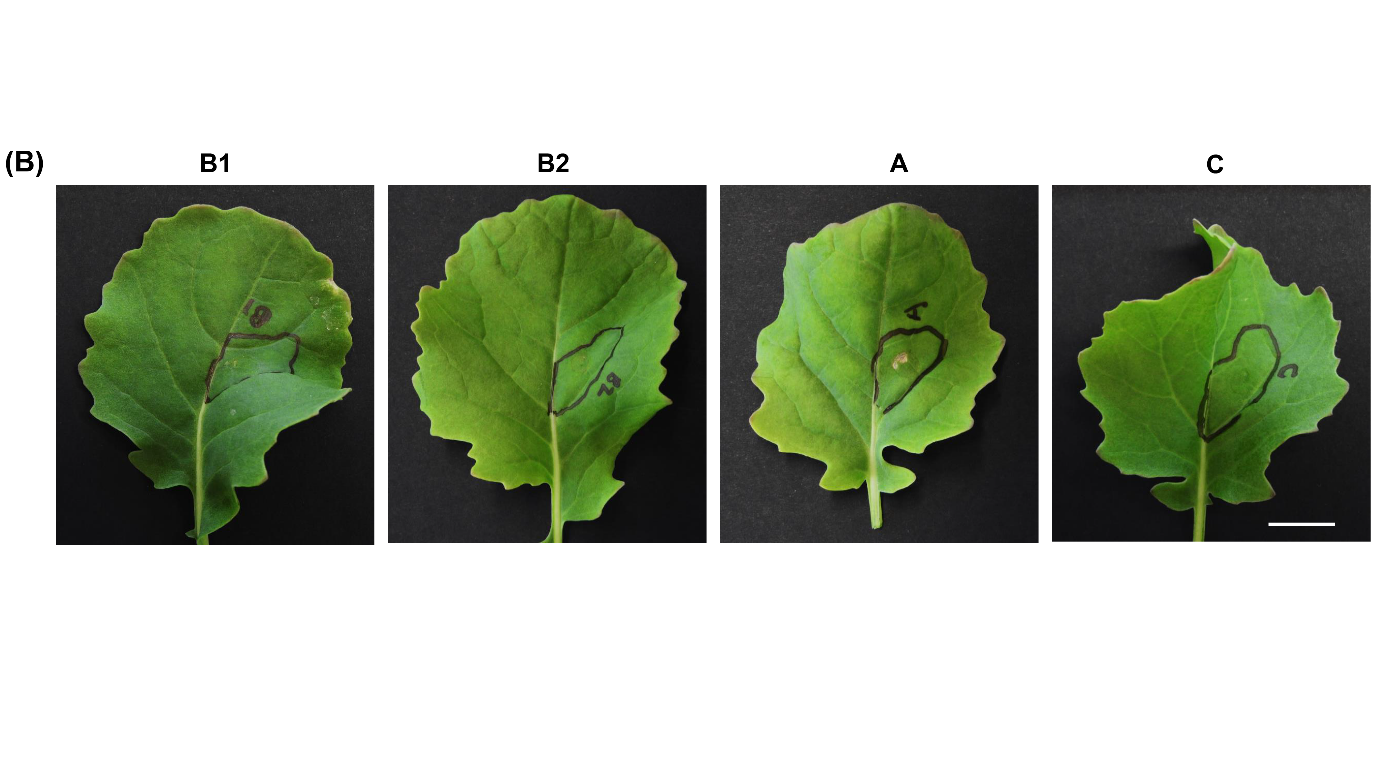


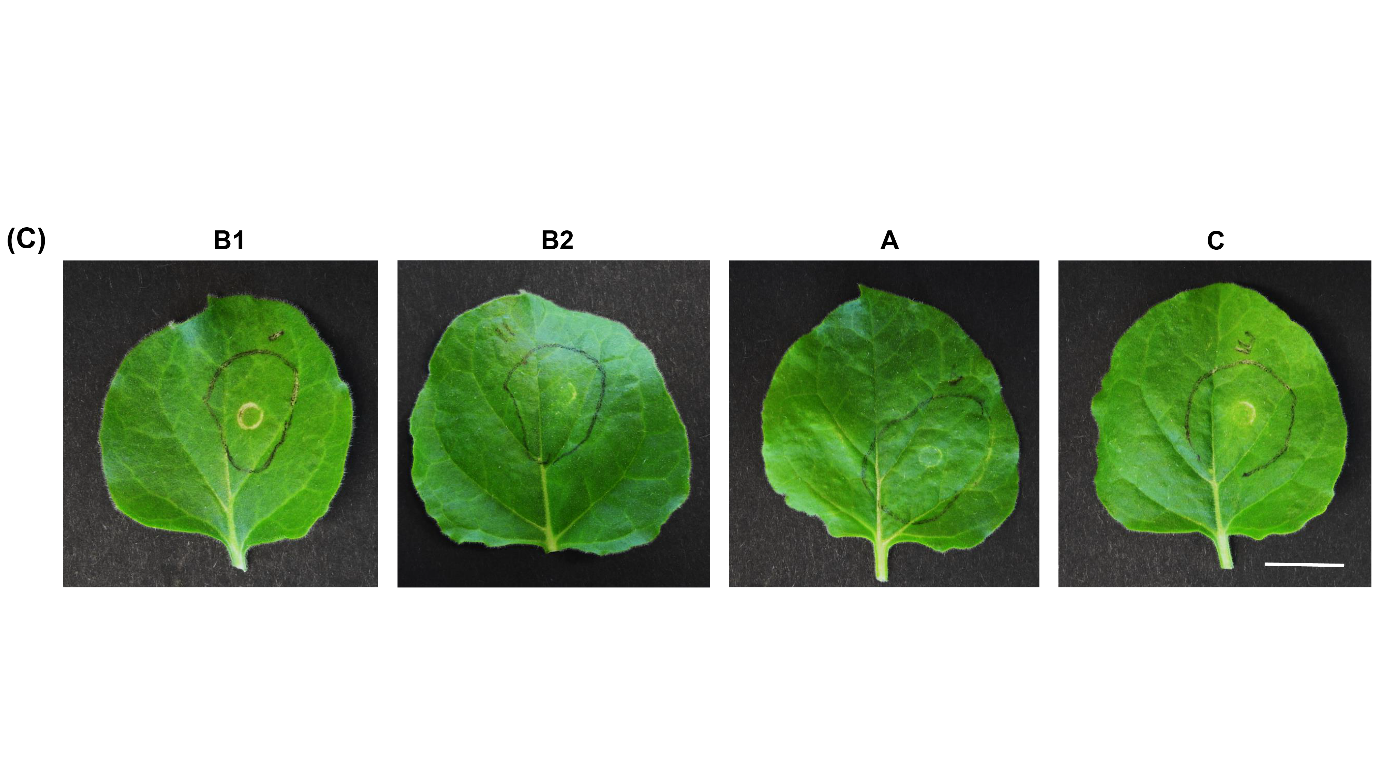


**Fig. S7.** Response of non-wheat monocot plants to *P. tritici-repentis* effectors. Leaves of ryegrass and *Brachypodium distachyon* were infiltrated with ToxB at the concentration of 500 ng/µl (B1) and 200 ng/µl (B2), *E. coli* crude cell extract containing ToxA (A) and empty vector as control (C). Annual and perennial varieties of ryegrass are indicated. Representative leaves were photographed 10 days post-infiltration. Scale bar, 10 cm.


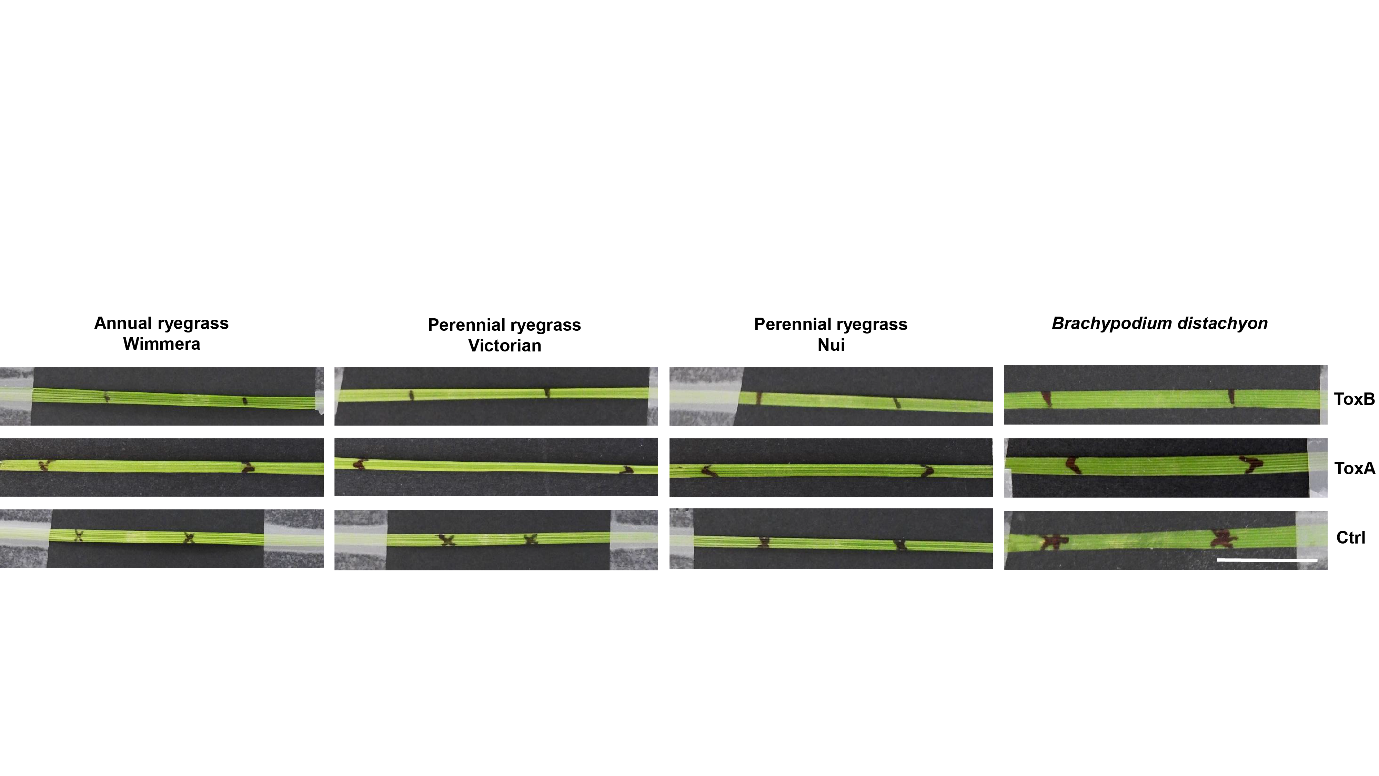

Supplement: Supplementary file 2 [file Table_2.docx]
